# The onco-functional reorganization of language network underlying metaplasticity induced by gliomas

**DOI:** 10.3389/fonc.2026.1850713

**Published:** 2026-05-29

**Authors:** Lu Jin, Zentao Zuo, Jie Kang, Fangzheng Liu, Yifan Song, Xin Liu, Kefan Cai, Wentao Wu, Chuzhong Li, Yazhuo Zhang, Songbai Gui

**Affiliations:** 1 Department of Neurosurgery, Beijing Tiantan Hospital, Capital Medical University, Beijing, China; 2 Beijing Neurosurgical Institute, Capital Medical University, Beijing, China; 3 State Key Laboratory of Brain and Cognitive Science, Institute of Biophysics, Chinese Academy of Sciences, Beijing, China; 4 Department of Otolaryngology, Head and Surgery, Beijing Children’s Hospital, Capital Medical University, National Center for Children’s Health, Beijing, China

**Keywords:** dynamic causal modelling, glioma, metaplasticity, network neurosurgery, onco-functional equilibrium, reorganized language network

## Abstract

**Background:**

Modern linguistic theories propose that language processing is supported by widely distributed large-scale modules that flexibly interact with domain-general networks. Progressive gliomas tend to reshape the language-associated systems causing dynamic reorganization that can be characterized by resting-state and task-based fMRI, thereby informing surgical decision-making. However, the interplay of neuropathological factors influencing reorganization patterns remains unclear, with ambiguity regarding the recruitment of cognitive resources across various levels of linguistic complexity.

**Methods:**

We included 100 patients with gliomas and 127 matched normal controls in this study. Activation analyses were performed on task fMRI data acquired during a picture-naming paradigm. Spectral DCM and connectome analyses were combined to examine state-dependent shifts between rest and task conditions. ANOVAs were used to assess relationships between clinicopathological factors and compensatory mechanisms. Mediation analyses were used to explore the mediated pathways among clinicopathological factors, topological indicators, and language performance.

**Results:**

First, we observed widely distributed activations associated with glioma-induced perilesional and remote reorganization patterns, exhibiting predominantly right-hemispheric lateralization in domain-general networks. VLSM analyses further showed that the spatial distribution of these activation clusters was significantly associated with tumor location and grade. Second, spectral DCM analyses indicated that task demands were associated with a greater number of positive effective connections across the reorganized language network, interactions among which were inherently dynamic varying with exogenous linguistic complexity and endogenous functional integrity. Third, ANOVAs suggested that alterations in language network topology were accompanied by flexible engagement of domain-general network components, which were associated with a partial restoration of the balance between integration and segregation under task conditions. Finally, we identified associations and potential mediation pathways among clinicopathological factors, topological properties, and language performance, consistent with the concept of glioma-related network metaplasticity.

**Conclusion:**

Our findings highlight that the dynamics of language reorganization depend on clinicopathological factors of gliomas and may thus open new perspectives for personalized surgical strategies for functional protection in the era of network neurosurgery. These group-level findings provide a modeling framework that may guide future research toward individualized network assessment and surgical planning.

## Introduction

Gliomas are the most common type of primary brain tumor; their progressive growth pattern can trigger large-scale functional reorganization, thereby inducing a spectrum of cognitive and linguistic compensation before surgery in contrast to acute lesions (such as traumatic brain injury, acute cerebral infarction, and hypertensive intracerebral hemorrhage) (1–4). Thus, patients with gliomas affecting language areas show considerable variability in preoperative manifestations and postoperative neurological recovery. However, the mechanisms underlying this process of functional reorganization remain unclear and require further investigation. Recent evidence from direct electrical stimulation and noninvasive functional neuroimaging studies has shown that numerous functional language areas connected by cortical–subcortical neural circuits underlie functional reorganization in response to both intrinsic pathological damage and external linguistic demands during the course of disease (5, 6). These findings regarding language compensation have been summarized into three established, non-mutually exclusive hypotheses. The first two are termed the “perilesional” (tissue around the lesion) and “laterality-shift” (contralateral homotopic cortex) hypotheses, which compensate for lesion-induced deficits in specialized linguistic functions (7, 8). The third hypothesis, in contrast, asserts a conflicting view that loss of transcallosal inhibition of the contralateral homotopic cortex hinders recovery (“disinhibition” hypothesis) (9–12). Those conflicting theories may create confusion regarding the development of surgical plans based on the latest concepts of brain metaplasticity and network neurosurgery, which advocate multistage therapeutic strategies to optimize both overall survival and quality of life for glioma patients (2, 13–17). Thus, a comprehensive mechanistic understanding of multi-scale language network reorganization underlying glioma-induced metaplasticity urgently needs to be clarified.

According to classical models, language processing has been recognized as a highly specialized and static framework, outlining a dual anatomical model of dorsal and ventral streams (18, 19). The ventral stream is largely bilaterally organized, including the posterior part of the superior temporal sulcus, the dorsolateral prefrontal cortex, and the pars orbitalis of the inferior frontal gyrus, which is involved in semantic processing and conceptual comprehension (20, 21). The dorsal stream is strongly left-hemisphere dominant and consists of inferior parietal lobe, the posterior part of the inferior temporal gyrus, and the pars opercularis of the inferior frontal gyrus (22, 23). This pathway is dedicated to articulatory representation and phonological working memory, operating in parallel to the semantic system supported by the ventral stream (24). Since the proposal of the brain connectome framework, recent studies have reframed these static anatomical streams into a large-scale functional network, placing particular emphasis on the characteristics of dynamical hierarchical interactions among different language functional modules (25). This new theory is based on the assumption that language processing not only depends on brain regions subserving specialized functions such as phonological, semantic, and syntactic representations but also is supported by the domain-general brain regions responsible for cognitive control, working memory, attention, and execution function (26–28). The typical networks engaged in linguistic tasks include the cingulo-opercular and fronto-parietal networks, which are usually depicted as functionally coactivated and spatially overlapping with language network in a task-dependent manner (29, 30). From this perspective, previously reported language-specific compensatory activation might reflect the upregulation of domain-general system activity to meet the growing cognitive demands imposed by linguistic complexity following damage to the downstream domain-specific language regions (6). However, how exactly the language-specific and domain-general components get dynamically involved to complete a task-oriented reconfiguration remains to be discovered.

In view of the mentioned issue about how the language network can be reorganized at the large-scale brain connectome level during the course of glioma, here we use the resting-state and naming-task fMRI data to explore the dynamic patterns and functional shifts under different mental states. By combining dynamic causal modeling (DCM) with connectome analysis, it can provide novel insights into understanding the compensation and recovery of language function at causal mechanistic and neurophysiological activity levels (31). Under the concept of metaplasticity, the effective connectivity constructed by DCM is capable of directly estimating the causal influence of neuroplastic functions by inferring the neuronal activity from the observed hemodynamic responses (32, 33). The following connectome model elucidates the topological properties of integration and segregation supporting the framework of abnormal onco-functional interactions during the progression of glioma (34–36). We hypothesized that the dysregulations between language-specific and domain-general communities are responsible for glioma-induced language deficits, the plasticity of which is mainly modulated through dynamic shifts in functional integration and segregation across adaptive meta-networks (37).

First, we identified the compensatory communities characterized by significantly increased activation clusters through a between-group comparison of task-based fMRI data. Then, we adopted three different lines for activation studies at cluster level, namely, (i) the activation patterns associated with clinicopathological factors, (ii) the spatial relationships between tumor sites and compensatory clusters, and (ii) the alterations in hemispheric lateralization for domain-general and language-specific networks. Second, we performed spectral DCM (spDCM) analyses to construct the effective connectivity of the reorganized language network (rLN) for both rest and task conditions. The weighted and directed matrices were entered into the parametric empirical Bayes (PEB) analysis, from which group commonalities and differences of intrinsic networks could be detected (38, 39). Then, three major questions at meta-network level were analyzed, namely, (i) the differences of binary effective connections between rest and task conditions, (ii) the connectivity patterns associated with clinicopathological factors, and (iii) the mechanisms of functional improvement of the rLN related to the compensatory community. Finally, to integrate the dynamics of glioma-related language reorganization, we analyzed the pairwise correlations and mediated effects among clinicopathological factors, behavior performance, and functional attributes across individual patients.

## Materials and methods

### Participants

**Figure 1:**
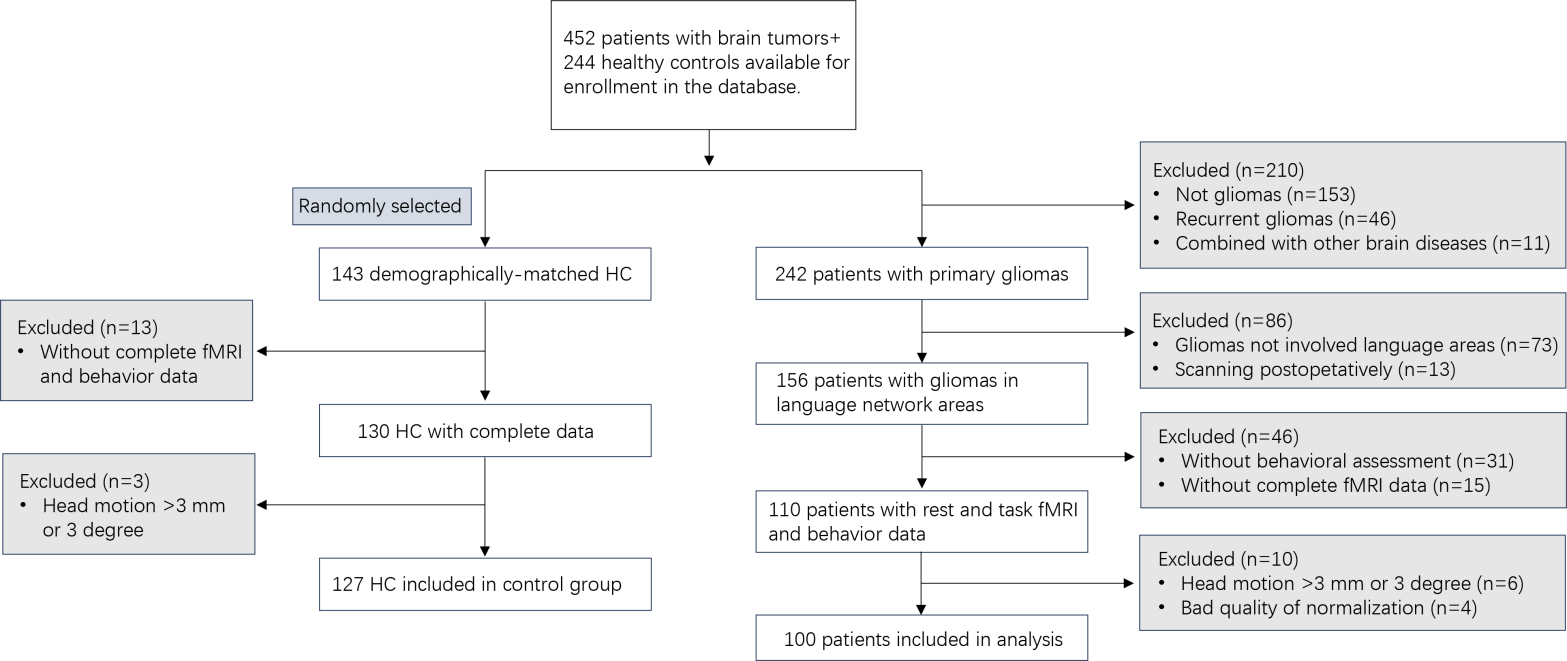
Exclusion flowchart of the patients and controls in this study.

A total of 452 hospitalized patients admitted to the Department of Neurosurgery at Beijing Tiantan Hospital were enrolled into a study database (Huaruihuinao Plat-form, Beijing Neurosurgical Institute, Capital Medical University, Beijing, China). Meanwhile, we recruited 244 healthy control volunteers from the community to complete the data collection schedule for the research project (The Construction of Fine Dynamic Map in Human Eloquent Brain Areas; no. Z171100000117002). Subjects were excluded from this project for any of the following reasons (1): contraindications for MRI examination including claustrophobia or scanner-incompatible implants (2), inability to finish experimental scanning paradigms (3), inability to complete the neurobiological behavioral assessments, and (4) without signature on the informed consent for this project. According to the purpose of this study, 242 patients with primary gliomas (PA) confirmed by pathological examination and 143 demographically matched normal controls (NC) were selected from the database. Further enrollment criteria included (1) age >14 and <80 years (2), gliomas affecting similar language functional regions defined by previous research (40, 41) (3), no symptoms of motor impairment (4), no history of brain traumatic injury and brain surgery (including needle biopsy), and (5) no history of neurological or psychiatric diseases. The exclusion flowchart of the patients and controls is presented in **Figure 1**.

All participants underwent a behavioral test battery that included measures of cognitive capability and neuropsychological status, as assessed using Mini Mental State Examination (MMSE; including orientation, memory, calculation, and language) and Karnofsky Performance Scale (KPS), respectively. The handedness preference was assessed using Edinburg Handedness Inventory (EHI). This study was approved by the ethics committee of Beijing Tiantan Hospital, Capital Medical University, with written informed consents obtained from all participants.

### Imaging acquisition

Whole-brain functional and structural MRI data were obtained using a Siemens Prisma 3.0 Tesla scanner (Siemens Healthineers, Erlangen, Germany) equipped with a 20-channel head coil. Three types of images were collected, namely, (i) high-resolution 3D T1-weighted images, (ii) resting-state fMRI images, and (iii) naming-task fMRI images. The 3D T1-weighted sagittal images were acquired using a magnetization-prepared rapid acquisition gradient echo (MPRAGE) sequence: 192 slices, voxel size = 1 × 1 × 1 mm, acquisition time = 7.4 min, acquisition matrix = 256 × 256, slice thickness/gap = 1/0 mm, TI/TR/TE = 900/2,300/2.3 ms, flip angle = 8°, field of view (FOV) = 256 × 256 mm^2^. The resting-state fMRI data were acquired using an echo-planar image (EPI) sequence: 30 axial slices, 200 scans, acquisition matrix = 64 × 64, slice thickness/gap = 5/0.5 mm, repetition time = 2,000 ms, echo time = 30 ms, acquisition time = 6.7 min, FOV = 192 × 192 mm^2^. All subjects were instructed to remain relax but awake and to fixate on a crosshair (“+”) presented at the center of the screen during resting-state scanning. For the naming task conditions, the parameter settings and acquisition time are the same as the resting-state fMRI images. The naming task was presented in a block design, and the entire task includes eight blocks. Each picture was presented for 2 s with a 1-s inter-stimulus interval, and nine pictures in total were presented within a 30-s block (3 s of instruction for preparation at the beginning of each block). These blocks were alternated repeatedly with the control blocks for the presentation of fixation (“+”) in the center of the screen with a duration of 20 s. A training session before formal scanning was conducted to ensure that the subjects fully understood the experimental design and performed correctly according to the instructions; the participants were instructed to whisper during naming to minimize head motion.

### Data processing and lesion mapping

**Figure 2:**
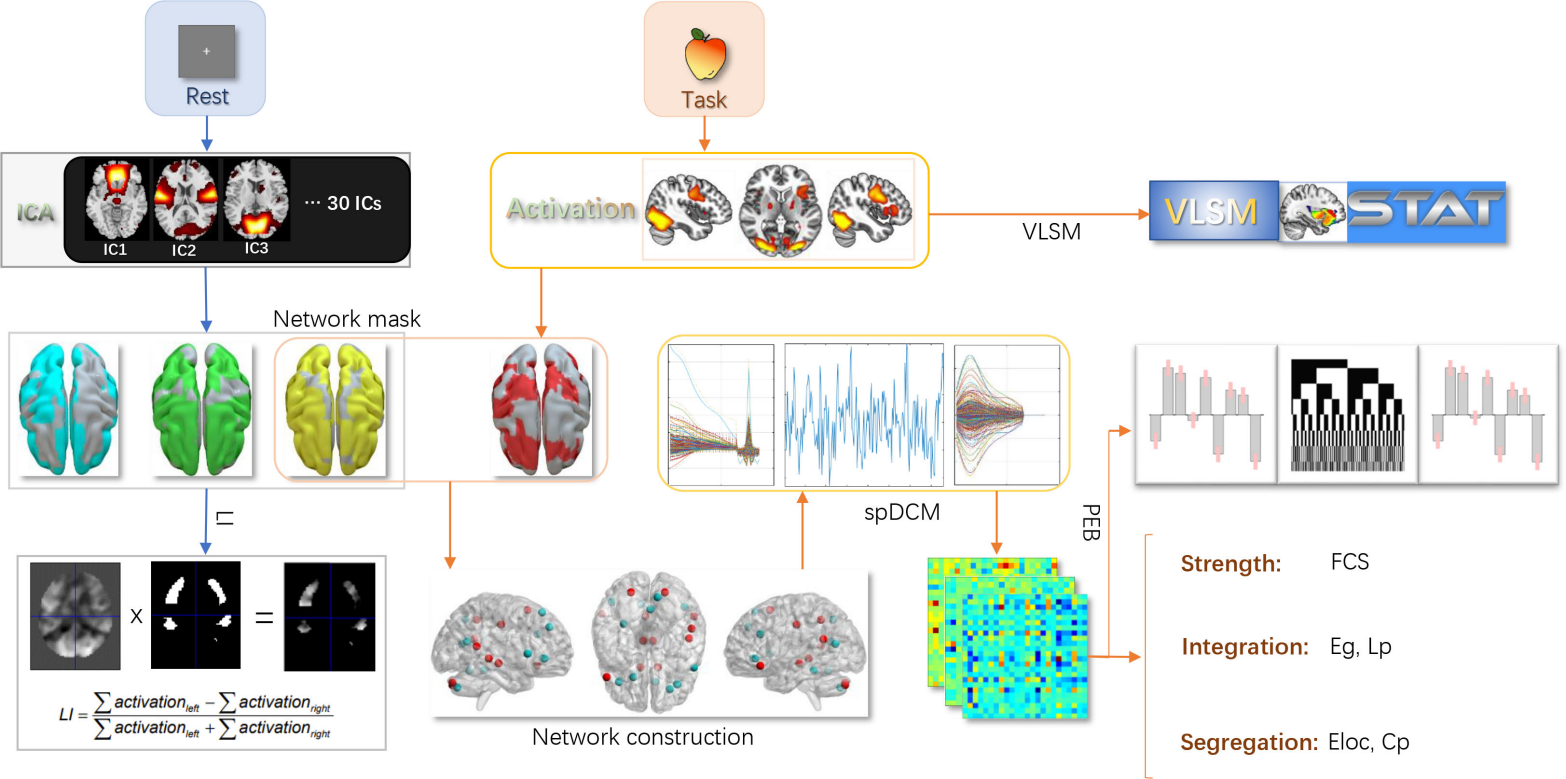
Overview of the study. Resting-state and task fMRI data were combined to construct the dynamic meta-network of glioma-induced neuroplasticity. Connectome analyses based on effective connections extracted from dynamic causal modeling (DCM) provide a dynamic interplay of functional connection strength (FCS), integration, and segregation between onco-functional metaplasticity at network level.

The flowchart of this study is shown in **Figure 2**. Both task and resting-state fMRI data were preprocessed using DPABI toolbox (http://www.rfmri.org/dpabi). In line with recent evidence that parsimonious motion correction is preferable in task-based fMRI, we employed the six-parameter rigid-body model for nuisance regression. A systematic comparison of motion correction strategies in clinical task-fMRI demonstrated that models using six motion parameters (MPs) outperform those using the Friston-24 parameter model, as the latter can overcorrect and inadvertently remove task-related blood oxygen level-dependent (BOLD) variance, thereby reducing sensitivity to true activations (42). This is particularly relevant for block-design naming tasks, where the evoked hemodynamic response is robust and structurally distinct from gradual head motion. Moreover, standard DCM and spectral DCM pipelines for task-based effective connectivity analyses conventionally use six MPs to preserve degrees of freedom while controlling for motion confounds (43, 44). In neuro-oncological imaging, aggressive nuisance regression is also avoided because higher-order expansions may capture variance related to tumor-induced field inhomogeneities rather than pure head motion (45). The preprocessing of the fMRI data included (i) slice timing correction for asynchronous acquisition time over slices in a volume, (ii) rigid-body registration to realign all images within a session to the first volume using a six-parameter transformation, (iii) six parameters of head motion obtained by rigid body correction for nuisance regression, (iv) individual 3D-T1 weighted images registered to the mean functional image using a rigid-body transformation, (v) segmentation of transformed structural images into gray matter, white matter, and cerebrospinal fluid (CSF), (vi) normalization of functional images into Montreal Neurological Institute (MNI) stereotactic standard space by an affine transformation and interpolation to a 3 × 3 × 3 mm cubic voxel size, (vii) smoothing the normalized functional images by convolution with a 6-mm full width at half-maximum Gaussian kernel in all directions, and (viii) quality control and normalization check to exclude excessive head motion (3.0 mm or 3.0° in maximum) and poorly normalized subjects.

**Figure 3:**
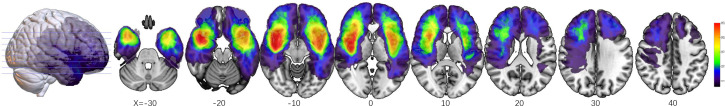
Lesion overlapping map for the cohort of glioma patients. False color and color bar represent the density of lesion distribution.

Additionally, for resting-state images, (i) the first 10 volumes were discarded to allow for saturation effects and magnetization equilibrium at the beginning of preprocessing and (ii) a temporal band-pass filtering (0.01–0.08 Hz) was applied to the time courses for independent component analysis (ICA). Specifically, for DCM and activation analysis, the regression of head motion was conducted during the process of first-level analysis in SPM12 (https://www.fil.ion.ucl.ac.uk/spm/) under MATLAB 2016a. To construct the lesion map, we first co-registered and normalized the T1 images to the Montreal Neurological Institute (MNI) template with resolution of 1 × 1 × 1 mm. Tumor masks were manually delineated on high-resolution T1-weighted images. Boundaries were validated against clinical pre-operative and post-operative contrast-enhanced T1 and FLAIR scans to exclude peritumoral edema. According to the “intratumoral functional regions” concept proposed by recent literatures, the masks encompassed the entire tumor volume, including solid tumor, necrosis, and cystic components, consistent with the surgical resection target (45, 46). Then, the individual tumor mask was stacked together to create the tumor overlapping map. The tumor overlapping map, reflecting the size and distribution of glioma at the overall group level, is shown in **Figure 3**. Finally, we used the MATLAB script “get_totals” provided by Ridgway (http://www.cs.ucl.ac.uk/staff/g.ridgway/vbm/get_totals.m.) to calculate each tumor volume (TV) in standard space.

### Group ICA analyses of resting-state fMRI

We performed ICA using the Group ICA toolbox (GIFT, https://trendscenter.org/software/gift/) by Infomax algorithm for resting-state images of all subjects. A total of 30 independent components (ICs) were decomposed into group-wide spatial maps of brain networks. The group mean spatial maps showing the largest and most significant spatial correlations with language network (LN), central executive network (CEN), and salience network (SN) in the resting-state networks template (https://findlab.stanford.edu/functional_ROIs.html) was automatically selected by the software and visually inspected as the aimed networks that were used for further analysis. Our primary consideration was to derive a set of common analytical templates directly from this dataset for downstream analyses while establishing consistent voxel-wise statistical comparison criteria across groups. The mean statistical network maps were generated at corrected *p* < 0.05 for multiple comparisons using a cluster-level family-wise error rate (FWE), with the cluster-forming (voxel-wise) threshold set at *p* < 0.001 for one-sample *t*-test analysis. The binary masks of SN, CEN, and LN were adopted as inclusive masks for laterality analysis and second-level activation analysis, and the identified peak voxel coordinates of LN were used for seed placement in the following DCM analysis.

### Compensatory activation analysis of task fMRI

#### First-level analysis

At the single-subject level, first-level linear regression analysis estimated the block of stimulus against rest (naming > rest). The block onsets and durations were modeled as stimulus functions. These event regressors were convolved with the canonical hemodynamic response function (HRF) to construct the model. The head motion parameters were used as regressors of no interest in the first-level model.

#### Second-level analysis

To determine the compensatory patterns of language activation, the individual first-level contrast maps for naming > rest were entered into a second-level analysis. We initialized a one-sample *t*-test to calculate the language-related activation maps for patients and controls separately (FWE correction: voxel-wise *p* < 0.001 and cluster-wise *p* < 0.05). Additionally, two-sample *t*-test general linear model (GLM) was used to explore the increased activations of patients compared to controls. The increased contrast analysis was restricted to language-related voxels using an inclusion mask derived from the combination of language-specific (LN) and domain-general network (CEN and SN) masks. Therefore, only voxels relevant to language processing were retained, which kept the searched map limited to the hypothetical brain areas of our study. To correct for multiple comparisons, inference of statistical significance was based on cluster extent threshold at *p* < 0.05 given a voxel-wise height threshold of *p* < 0.001 for false discovery rate (FDR), with a cluster size *k >*30 voxels. After completing the second-level statistical analysis for task data, we performed 2 × 2 × 2 mixed-design ANOVAs to assess the relationships between clinicopathological factors (hemisphere: left/right; lobe: frontal/temporal; grade: high/low) and compensatory activations in patients. The compensatory activations were extracted from spherical ROIs (*r* = 4 mm, sizes = 7 voxels) centered in the significantly increased clusters, which was obtained from the group comparison between patients and controls.

### Voxel lesion symptom mapping analysis

To clarify the directional relationships between glioma-induced perilesional and remote increased activations and tumor location in the naming task condition, we performed voxel lesion symptom mapping (VLSM) analysis using NiiStat (https://github.com/neurolabusc/NiiStat), a Matlab software package that has the advantage to examine at both voxel-wise and region-wise levels. We used NiiStat univariate analysis to explore significant correlations between the mean *T*-value in each compensatory cluster and binary glioma masks using general linear regression, with the Freedman–Lane permutation test at *p* < 0.05. The settings of 2,000 permutations and one minimum overlap in one area were adopted. We performed the VLSM analysis in a separate hemisphere, and both voxel-wise and region-wise (defined by AAL atlas) methods were used for statistical power and verifications.

### Network-based laterality index analysis

The laterality index (LI) relies on the basic computation LI = (left - right)/(left + right), which was calculated using the LI toolbox in conjunction with SPM12. The masks of language-specific network (LN) and domain-general networks (SN and CEN) identified using ICA were adopted as inclusive masks for lateralization calculation. Mean LI values were determined for language-related network masks of each individual after sampling across 20 different activation thresholds using the bootstrap method. Additionally, for regional literality assessment, the language-related masks were divided into subsystems of frontal, temporal, and parietal lobes, which were also estimated using the same bootstrap procedure. This algorithm yields a series of continuous variables of lateralization indices between -1 (indicating complete right-hemispheric dominance) and 1 (indicating complete left-hemispheric dominance). In the following analysis, bilateral language distribution was defined as LI from -0.2 to 0.2, left dominance as LI >0.2, and right dominance as LI <-0.2 consistent with laterality index cutoff values commonly used in language functional activation studies (47, 48).

### Spectral DCM analyses with PEB

**Figure 4:**
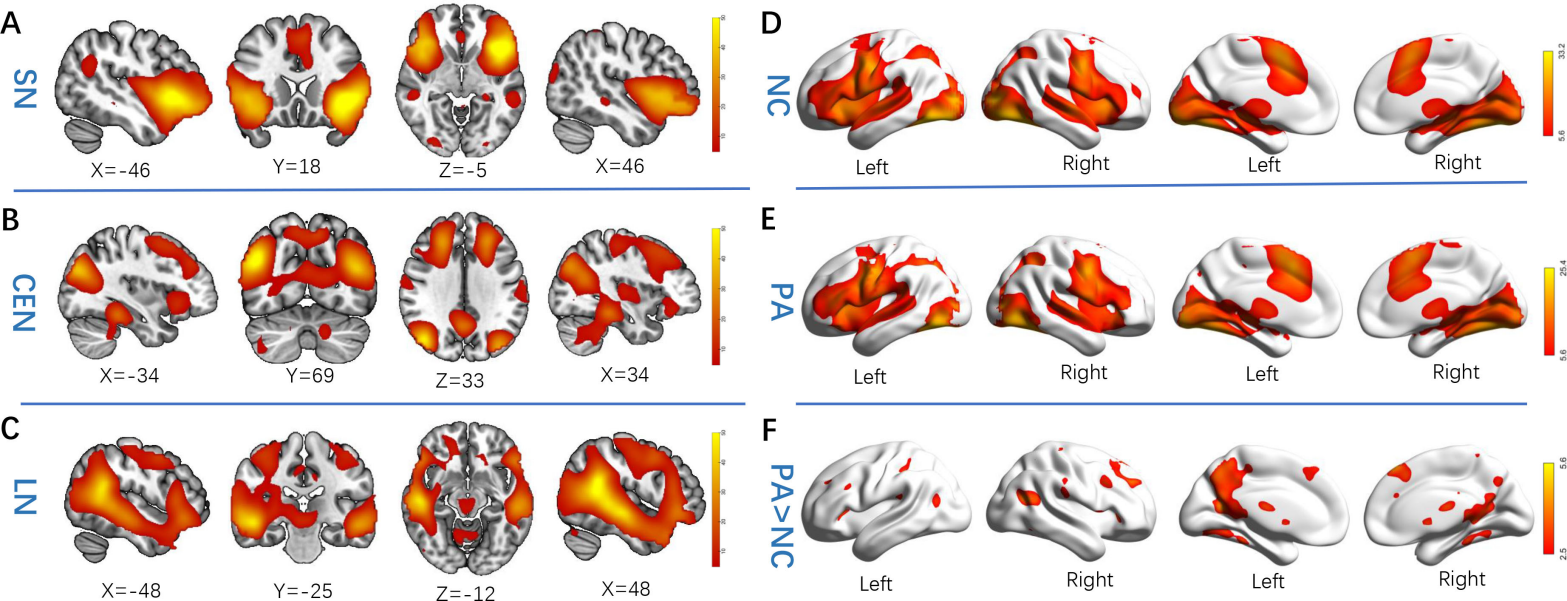
Language-related network and activation maps. Profiles of domain-general **(A, B)** and language-specific **(C)** network identified by ICA (*p* < 0.05, FWE-corrected for multiple comparisons at the cluster level). Patterns of naming activation in controls **(D)** and patients **(E)** (*p* < 0.05, FWE-corrected for multiple comparisons at the cluster level). **(F)** Compensatory map of increased activation clusters (*p* < 0.05, FDR-corrected for multiple comparisons at the cluster level).

We introduced the concept of reorganized LN (rLN) to study the functional shifts of metaplasticity under different task-load demands according to the linguistic theory of network neuroscience (49, 50). The rLN is composed of core community (Ccore) of language-specific network (**Figures 4C**, LN) identified using ICA and compensatory community (Ccom) detected using two-sample activation *T*-test between PA and NC (**Figure 4F**, PA > NC). Each of the community includes 12 ROIs with a 6-mm spherical radius. The peak MNI coordinates are listed in **Table 1** and shown in **Supplementary Figure 1**.

**Table 1:** Region and peak MNI coordinates of reorganized language network.

No.	Core community			No.	Compensatory community		
	Region	Side	MNI coordinate		Region	Side	MNI coordinate
1	SM-7	Left	-57 -51 21	1	Ang-3	Right	45 -60 18
2	Prec-4	Left	-3 -48 45	2	SF-2	Right	9 33 54
3	IF-tir-1	Left	-51 27 -12	3	SF-3	Right	27 42 36
4	Cre-crus-2	Left	-21 -78 -36	4	IF-tir-1	Right	36 36 -3
5	SMA-2	Left	-9 15 57	5	Cre-8	Right	12 -72 -39
6	MC-3	Left	-6 -18 39	6	AI-4	Right	57 15 3
7	ST-4	Right	57 -48 12	7	Ver-4-5	Right	3 -57 -18
8	ST-3	Right	54 -33 -3	8	AI-4	Left	-36 33 -3
9	ST-2	Right	51 -21 -9	9	Prec-4	Left	-9 -63 45
10	SMA-2	Right	12 15 57	10	SF-3	Left	-18 27 27
11	MC-3	Right	6 -18 39	11	PH-2	Left	-30 -39 -18
12	Cre-crus-1	Right	21 -78 -33	12	MO-3	Left	-45 -66 15

SM, superior marginal gyrus; Prec, precuneus area; IF-tri, pars triangularis of inferior frontal gyrus; Cre-crus, crebellum crus; SMA, supplementary motor area; MC, middle part of cingulum gyrus; ST, superior temporal gyrus; Ang, angular; SF, superior frontal gyrus; Cre, cerebellum; AI, anterior insula; Ver, vermis; SF, superior frontal gyrus; PH, parahippocampal gyrus; MO, middle occipital gyrus.

#### First-level analysis

We used spDCM with a fully connected model, generating a total of 576 effective connectivity parameters, for each subject at first-level analysis. The mean timeseries of 24 spherical ROIs of the rLN was extracted separately for both rest and task states and then preprocessed and partitioned into neural and non-neural (hemodynamic and noise) sources. The neural model of DCM captures directed hidden interactions between brain regions, with connection strength encoded in the matrices of the parameters. Parameter matrix A described the average effective connectivity (effect size) across experimental conditions for task fMRI and the average intrinsic connectivity of neuronal fluctuations under resting states (51). spDCM estimates the parameters of cross-correlation functions or cross-spectra of fMRI data by replacing the original time series (time-domain) with their second-order statistics (frequency-domain). By modeling the data features in terms of spectral power using the cross-spectral density (CSD) method, spDCM becomes quicker, more efficient, and more sensitive for comparing intrinsic effective connectivity between groups (52).

#### Second-level analysis

After modeling the effective connectivity of each subject’s time series using spDCM in the first-level analysis, the subject-specific connectivity parameters were entered into the group-level analysis using the PEB framework, where the commonality and difference across subjects are assessed and quantified with two regressors: the first modeling the group average commonality and the second modeling the group difference (53). PEB calculates the between-group variability using the GLM algorithm, which divides between-subject variability into design effect and additive random effect. The algorithm optimizes the reduced models from the full model by shrinking the priors of parameters to zero using Bayesian model reduction (BMR). To focus the results just on those parameters that had the greatest evidence across reduced models, we threshold the free energy at 95% posterior probability to compute the Bayesian model average (BMA)—the average of parameters from all reduced winner models weighted by their posterior probabilities. The free energy in the PEB analysis is the sum of accuracies minus the complexity induced by the GLM algorithm (54).

### Dynamic network topological properties calculations

We next tested the functional reconfiguration of the rLN from resting state to task conditions addressing two major questions, namely, (i) the topological alterations in network properties under the attack of gliomas and (ii) the onco-functional interplays invoked by linguistic complexity. For each participant, the topological properties of the rLN can be examined using a rich array of matrices provided by the general framework of graph theory. In this study, we investigated the topological properties of directed and weighted effective connections constructed using spDCM. To define the network edges, we selected the threshold of 95% free energy in the PEB analysis for the commonalities of patients and controls, as well as for the differences of patients compared to controls. Such threshold selection ensures consistence in the number of binary effective connections compared between groups. Moreover, a stricter threshold can reduce the risk of false-positive connections due to noise and offer a more accurate and lesser complex explanation for the data. All network analyses were performed using the GRETNA software (http://www.nitrc.org/projects/gretna/). Network characteristics can be generally classified into measures covering four aspects, namely, (i) functional connection strength (FCS), (ii) functional segregation, (iii) functional integration, and (iv) inter-community connection strength. The FCS is the mean of the sum weighted effective connection strength across all edges within the network or community. Measures of segregation are commonly based on the clustering coefficient (Cp) and local efficiency (Eloc). Measures of integration include the characteristic path length (Lp) and global efficiency (Eg). Detailed descriptions are presented in the **Supplementary Materials**.

### Correlation and mediation analyses

To examine potential treatment implications under the latest concept of network neurosurgery, we calculated the pair-wise correlations among clinicopathological factors, topological indicators, and behavior performances across patients. Pearson correlation analyses were performed across the main clinicopathological features (grade, volume, and duration), significant topological indicators (FCS, Eg, Lp, and Eloc), and behavior performances (MMSE, language). To dissect how onco-functional metaplasticity modifies the rLN dynamics, we constructed combined mediation models under both rest and task conditions. The glioma–connectome–behavior pathway linking tumor factors to language mediated by glioma–connectome equilibrium was constructed using PROCESS macro version 3.5 written by Andrew F. Hayes, which enabled us to simultaneously evaluate the direct and indirect mediation effects. Bootstrap simulation (*n* = 5,000) was set among all possible correlations of the three aspects, while age and sex were used as covariates of no interest in the models.

### Statistical and mediation analyses

Statistical analyses were conducted using IBM SPSS v23, with the significance level set at *P* < 0.05 for all tests after correction for multiple comparisons. For the activation analyses, to estimate the significant main and interaction effects across clinicopathological factors of gliomas, mixed-design ANOVAs were used for group comparisons. For laterality index analyses, between-group differences in continuous lateralization indices were compared by means of two-sample *t*-test. Categorical variables of language activation dominance were compared using chi-square (*χ*^2^) analyses. For network analyses, to determine the within- and between-subject effects of patients and controls, a repeated-measures ANOVA was performed on each network parameter.

### Visualization of results

Significant fMRI activation maps were overlaid on a standard brain (MNI-152 template) using the MRIcroGL_windows tool (https://github.com/synopse/mORMot). ICA surface maps were overlaid on a standard brain (MNI-152 template) using the MRIcroS tool (https://www.nitrc.org : MRIcroS: Tool/Resource Info). Graph metrics of spDCM networks were displayed using MATLAB 2016a (Mathworks, Natick, MA, USA). The commonalities and differences of the PEB results were portrayed using Circos (http://www.circos.ca/). GraphPad Prism version 8 (GraphPad Software, La Jolla, CA, USA) was used to generate graphs and scatter plots.

## Results

### Clinical and demographic characteristics

After exclusion for excessive head motion (>3.0 mm or >3.0°), a total of 100 glioma patients and 127 healthy controls were included in this analysis. No significant differences were found in age, sex, education, or handedness between the NC and PA groups. The demographic information of the cohort is presented in **Table 2**. Specifically, the patients were classified into subgroups based on three glioma-related factors [hemisphere (left/right, 47/53), lobe (frontal/temporal, 56/43), and grade (low/high, 47/53)] for further VLSM and ANOVA analyses. Significant behavioral differences between PA and NC were observed for MMSE (*T* = 7.83, *p* < 0.001), calculation (*T* = 4.86, *p* < 0.001), language (*T* = 9.03, *p* < 0.001), and KPS (*T* = 18.98, *p* < 0.001), but not for memory or orientation (*T* = 2.39, *p* = 0.18).

**Table 2:** Demographic and clinical characteristics for glioma patients and healthy controls.

Variables	NC (127)	PA (100)	*F*/*T*/*χ*^2^	*p*
General information				
Age (mean ± SD)	42.7 ± 10.9	43.7 ± 12.3	3.10	0.55
Gender (female/male)	67/60	47/53	0.86	0.39
Education (mean ± SD)	14.9 ± 3.4	11.6 ± 3.8	6.79	0.54
EHI (L/B/R)	2/2/123	1/4/95	1.41	0.50
Symptom duration	–	6.0 ± 8.5	–	–
Clinicopathological factors				
Hemisphere (left/right)	–	47/53	–	–
Lobe (frontal/temporal)	–	56/43	–	–
Grade (low/high)	–	47/53	–	–
Behavioral assessments				
MMSE (mean ± SD)	29.7 ± 0.8	27.7 ± 2.7	7.83	**<0.001**
Orientation (0–10)	9.8 ± 0.6	9.5 ± 1.1	2.39	0.18
Memory (0–3)	3.0 ± 0.0	3.0 ± 0.0	–	–
Calculation (0–5)	4.9 ± 0.3	4.4 ± 1.2	4.86	**<0.001**
Language (0–12)	12.0 ± 0.0	10.8 ± 1.5	9.03	**<0.001**
KPS (mean ± SD)	100.0 ± 0.0	84.6 ± 9.1	18.98	**<0.001**

NC, normal control; PA, patient; EHI (L/B/R), Edinburgh handedness inventory (left/bilateral/right); KPS, Karnofsky Performance Scale.

The bold values indicate statistically significant p-values.

### Network-based functional activation

Among the 30 decomposed ICs for resting-state fMRI from all subjects, the group ICA identified domain-general networks of SN in IC14 (**Figure 4A**; **Supplementary Table 1**) and CEN in IC19 (**Figure 4B**; **Supplementary Table 2**), while the language-specific network of LN was found in IC29 (**Figure 4C**; **Supplementary Table 3**). Significant activation clusters are shown in **Figure 4D** for NC and **Figure 4E** for PA in the single-group analyses of naming task fMRI (FWE-corrected, *p* < 0.001). Then, in the comparison between groups, PA showed a significantly increased activation in the brain areas within widely distributed domain-general networks compared to NC, mainly including the right anterior insula, the left inferior frontal gyrus, the bilateral precuneus, the medial part of the bilateral superior frontal gyrus, the posterior part of the bilateral middle temporal gyrus, the bilateral inferior temporal gyrus, and the left angular gyrus (**Figure 4F**; **Table 3**, FDR-corrected, *p* < 0.05), while, interestingly, there was no significantly reduced activation in the opposite comparison (FDR-corrected, *p* < 0.05).

**Table 3:** Clusters with increased activation of PA than NC in the comparison of naming task fMRI data (FDR-corrected, *p* < 0.05).

Regions (AICHA)	Hemisphere	Peak coordinates (MNI)	Statistics			
			*Q*_FDR_	*T*	*P*_uncoor_	*k*
Insula-anterior-4	Left	-30 27 9	0.016	4.65	<0.001	72
Inf-frontal-tri-1	Left	-36 33 -3	0.018	3.88	<0.001	Subcluster
Precuneus-1	Left	-21 -60 15	0.016	4.49	<0.001	760
Precuneus-9	Left	-9 -63 45	0.016	4.31	<0.001	Subcluster
Precuneus-3	Left	-15 -51 42	0.018	3.89	<0.001	Subcluster
Sup-frontal-3	Left	-18 27 27	0.018	3.75	<0.001	258
Sup-frontal-4	Left	-15 24 36	0.018	3.66	<0.001	Subcluster
Inf-frontal-2	Left	-27 27 18	0.024	3.23	0.001	Subcluster
Fusiform-3	Left	-45 -39 -15	0.018	3.64	<0.001	57
Parahippocampal-2	Left	-30 -39 -18	0.018	3.61	<0.001	Subcluster
Fusiform-5	Left	-30 -48 -15	0.041	2.77	0.003	Subcluster
Vermis-4-5	Left	0 -57 -18	0.019	3.42	<0.001	83
Angular-3	Left	-51 -72 18	0.025	3.18	0.001	69
Mid-Occipital-3	Left	-45 -66 15	0.028	3.09	0.001	Subcluster
Inf-Temporal-5	Left	-42 -66 6	0.028	3.09	0.001	Subcluster
Fusiform-4	Right	42 -42 -12	<0.001	5.69	<0.001	1,063
Angular-3	Right	45 -60 18	0.007	4.44	<0.001	Subcluster
Parietooccipital-1	Right	18 -45 6	0.007	4.11	<0.001	Subcluster
Sup-Frontal-2	Right	9 33 54	0.005	4.65	<0.001	792
Sup-Frontal-3	Right	18 24 33	0.006	4.55	<0.001	Subcluster
Sup-Frontal-4	Right	27 42 36	0.007	4.27	<0.001	Subcluster
Inf-Frontal-tri-1	Right	36 36 -3	0.007	4.24	<0.001	151
Insula-anterior-3	Right	33 30 6	0.007	4.13	<0.001	Subcluster
Cerebellum-8	Right	12 -72 -39	0.011	3.66	<0.001	39
Cerebellum-8	Right	24 -66 -45	0.042	2.62	<0.001	Subcluster
Insula-anterior-4	Right	57 15 3	0.013	3.58	<0.001	57
Lingual-2	Right	18 -57 -15	0.019	3.21	0.001	Subcluster
Lingual-4	Right	9 -66 -12	0.037	2.71	0.004	Subcluster
Postcentral-2	Right	36 -27 45	0.024	3.07	0.001	57

### Association between greater activation clusters and tumor sites

**Figure 5:**
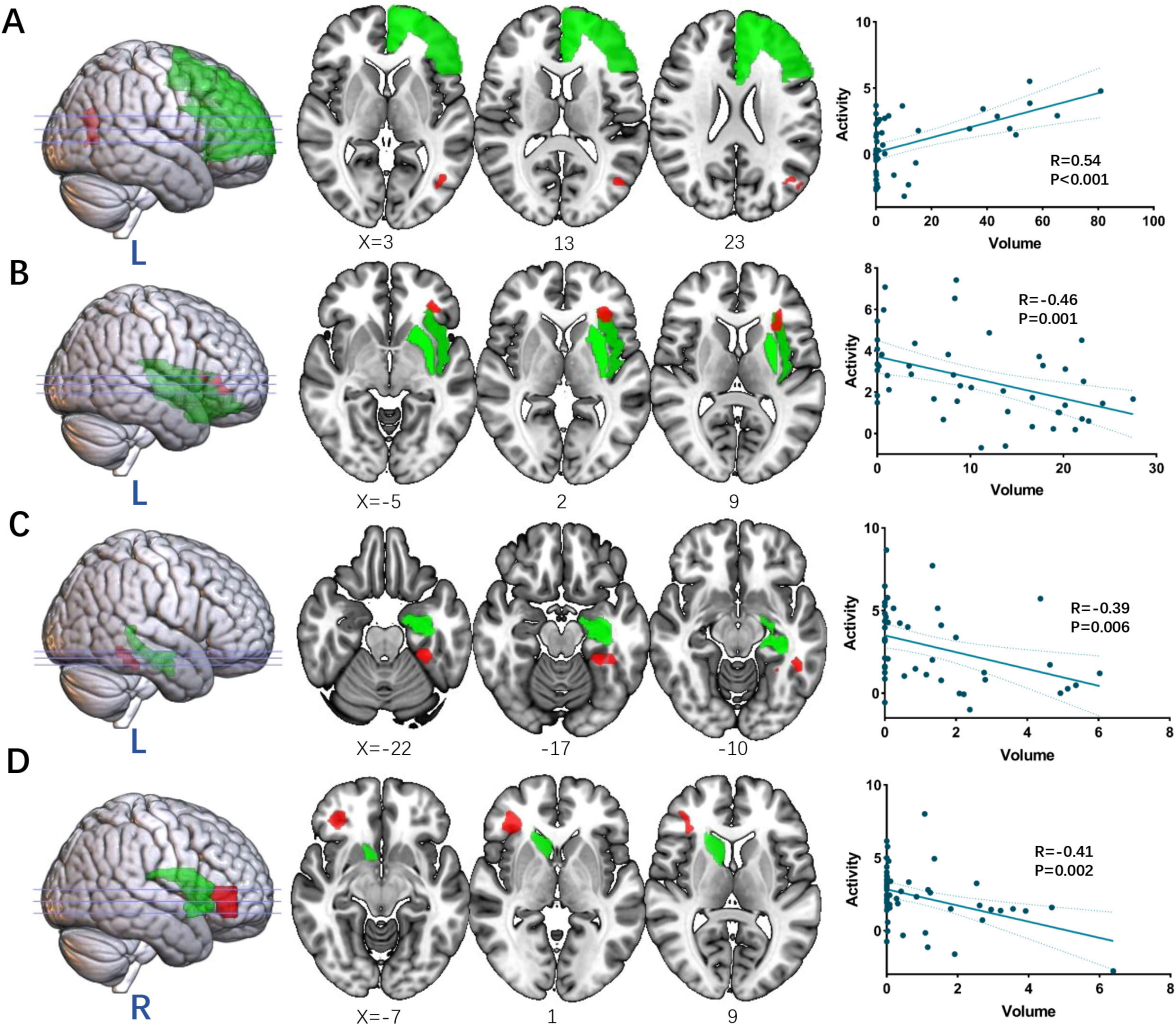
The remote **(A)** and perilesional **(B–D)** compensatory reorientation associated with lesion sites in naming task condition. As the tumor volume increases, the compensatory capacity of the peritumoral brain tissue gradually weakens, while the functional activities of the distant brain regions become enhanced. The green brain regions represent the location of the tumor, and the red activation clusters represent the compensatory brain areas detected by activation analysis. L, left hemisphere; R, right hemisphere.

A positive association between lesion sites extending from the superior frontal gyrus and the anterior cingulum to the inferior temporal gyrus and greater activation clusters, including the angular gyrus and posterior part of the inferior temporal gyrus, was identified as a remote neuroplastic pattern within the left hemisphere (**Figure 5A**, *R* = 0.54, *p* < 0.001). Tumors involving the orbital part of the inferior frontal gyrus, anterior insula, and putamen in the left hemisphere have a remarkable statistical association with the adjacent clusters of the anterior insula and pars triangularis of the inferior frontal gyrus, with a significantly negative correlation between the lesion volume and the activation value (**Figure 5B**, *R* = -0.46, *p* = 0.001). Another significantly negative association in the left hemisphere was found between the tumor-involved parahippocampal gyrus/hippocampus and the perilesional compensatory fusiform cluster (**Figure 5C**, *R* = -0.39, *p* = 0.006). In the right hemisphere, a negative association was also found between the lesioned caudate nucleus and the compensatory cluster comprising the pars triangularis of the inferior frontal gyrus and the anterior insula (**Figure 5D**, *R* = -0.41, *p* = 0.002) (**Table 4**). These results may indicate a directional relationship between the activity of greater activation clusters and the brain regions where gliomas grow at group level.

**Table 4:** Significant associations between the compensatory clusters and lesioned brain areas in the VLSM analysis.

Compensatory cluster (AICHA)	Cluster sides	Cluster sizes (voxels)	Tumor area (AAL)	Tumor side	Area size (voxels)	*Z*-score	Validation	
							*R*	*P*
Cluster-1	Left	69		Left	367,104		0.54	<0.001
Angular-3			Frontal-sup			3.67		
Occipital-mid-3			Frontal-mid			3.28		
Temporal-inf-5			Frontal-inf-tri			3.16		
			Frontal-sup-med			3.53		
			Cingulum-ant			3.58		
Cluster-2	Left	72		Left	90,721		-0.46	0.001
Insula-anterior-4			Frontal-inf-orb			-2.60		
Frontal-inf-tri-1			Insula			-2.59		
			Putamen			-3.26		
Cluster-3	Left	57		Left	19,710		-0.39	0.006
Fusiform-3			Hippocampus			-2.75		
Parahippocampal-2								
Fusiform-5								
Cluster-4	Right	151		Right	25,345		-0.41	0.002
Frontal-inf-tri-1			Caudate			-3.06		
Insula-anterior-3								

### Tumor pathology affecting compensatory activation and lateralization

**Figure 6:**
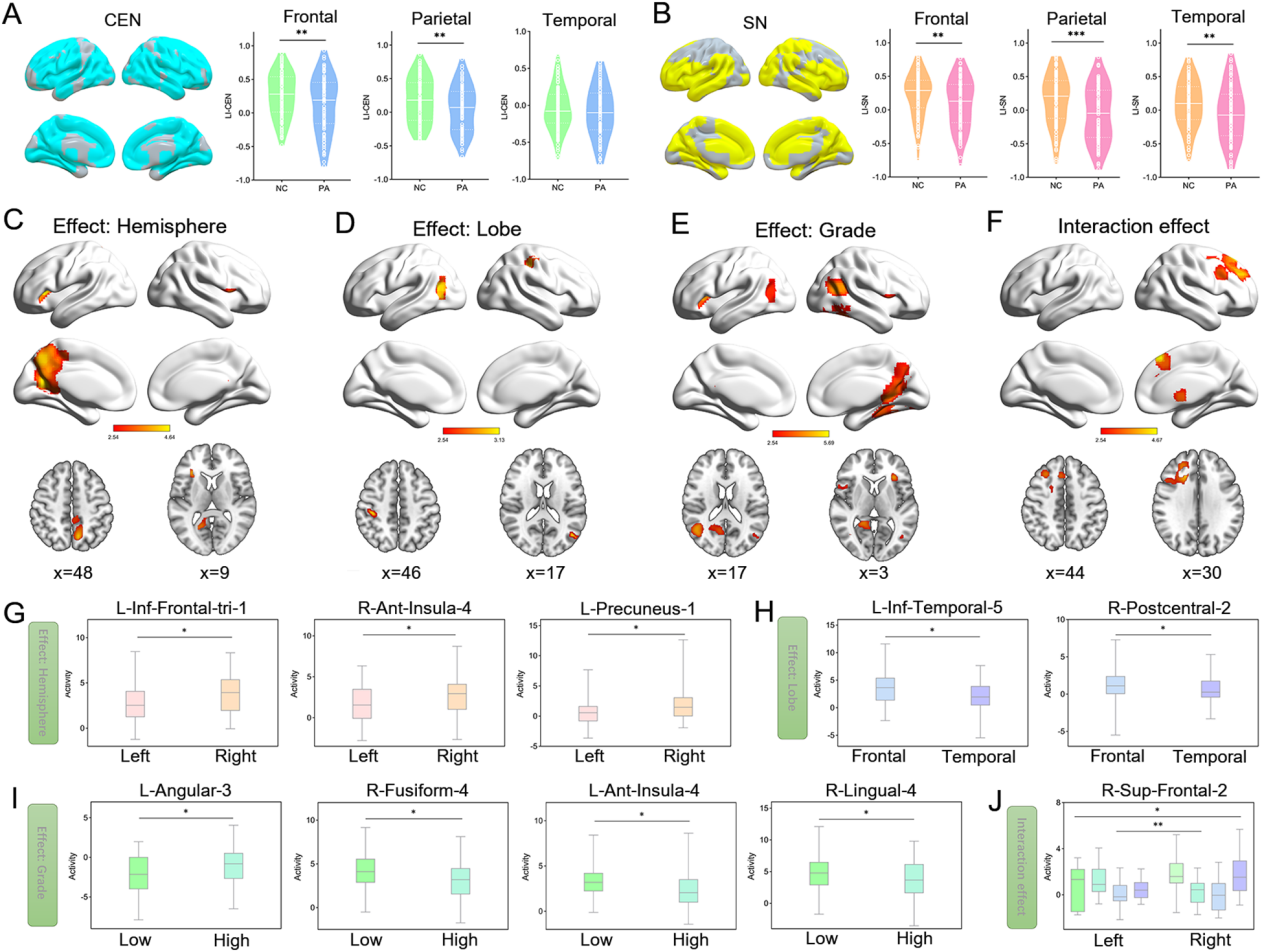
Activation analyses for glioma-induced metaplasticity associated with tumor pathological factors. **(A, B)** LI analyses for domain-general network. Systems in the frontal and parietal lobes of CEN **(A)** and systems in the frontal, parietal, and temporal lobes of SN **(B)** showed significant right-hemispheric lateralization. **(C–J)** Compensatory activation patterns associated with clinicopathological factors. **(C, G)** Main effect of hemisphere. **(D, H)** Main effect of lobe. **(E, I)** Main effect of grade. **(F, J)** Interaction effect of hemisphere × lobe × grade. Brain areas are localized in Atlas AICHA (Joliot et al., 2015). *p < 0.05, **p < 0.01, ***p < 0.001, FDR-corrected for multiple comparison, *t* > 2.54. LI, laterality index; CEN, central executive network; SN, salience.

For the lateralization analyses, the laterality index of LN showed no significant differences between PA and NC at its subnetwork systems within the frontal (LN-F: *p* = 0.24), parietal (LN-P: *p* = 0.40), and temporal (LN-T: *p* = 0.10) lobes (**Supplementary Table 4**). However, for domain-general masks, PA exhibited significant right-predominant activations in the CEN (**Figures 6A**, CEN-F: *p* = 0.004; CEN-P: *p* = 0.007) and SN (**Figures 6B**, SN-F: *p* = 0.006; SN-P: *p* < 0.001; SN-T: *p* = 0.005), except for CEN-T (CEN-T: *p* = 0.39). The binarized lateralization patterns according to categorical classification demonstrated similar results for the masks of LN, SN, and CEN, suggesting that PA showed higher domain-general activity presenting as right-hemispheric shifts during task conditions (**Supplementary Table 5**, LN-F: *p* = 0.24, LN-P: *p* = 0.65, LN-T: *p* = 0.47; CEN-F: *p* < 0.001, CEN-P: *p* = 0.014, CEN-T: *p* = 0.24; SN-F: *p* = 0.035; SN-P: *p* < 0.001; SN-T: *p* = 0.021).

Mixed-design ANOVAs revealed that clusters mainly associated with hemisphere, irrespective of lobe and grade, were identified in the right anterior insula [*F* (1,92) = 4.03 *p* = 0.048], the pars triangularis of the left inferior frontal gyrus [*F* (1,92) = 3.99 *p* = 0.049], and the left precuneus [*F* (1,92) = 4.77, *p* = 0.032] (**Figures 6C,G**). Activations in the regions of the right postcentral sulcus [*F* (1,92) = 4.18, *p* = 0.044)] and the left inferior temporal gyrus [*F* (1,92) = 4.67, *p* = 0.033] were independently associated with factor of lobe (**Figures 6D,H**). Activations in the regions of the right fusiform gyrus [*F*(1,92) = 5.12, *p* = 0.026], the right lingual gyrus [*F*(1,92) = 3.94, *p* = 0.050], the left anterior insula [*F*(1,92) = 4.45, *p* = 0.038], and the left angular gyrus [*F*(1,92) = 4.70, *p* = 0.033] showed a remarkable association with tumor grade (**Figures 6E,I**). Specifically, these significant main effects of clusters indicate compensatory brain areas accounting for specific clinicopathological factors of gliomas. Besides that, we also identified a lobe × grade × hemisphere interaction for the right superior frontal gyrus [*F*(1,92) = 6.34, *p* = 0.014] (**Figures 6F,J**). The right hemispheric interactions may indicate that the complex functional reorganization across various clinicopathological factors is primarily supported by the right cerebral areas, which is consistent with the findings of the lateralization analyses.

### Dynamic functional shifts under metaplasticity of the rLN

**Figure 7:**
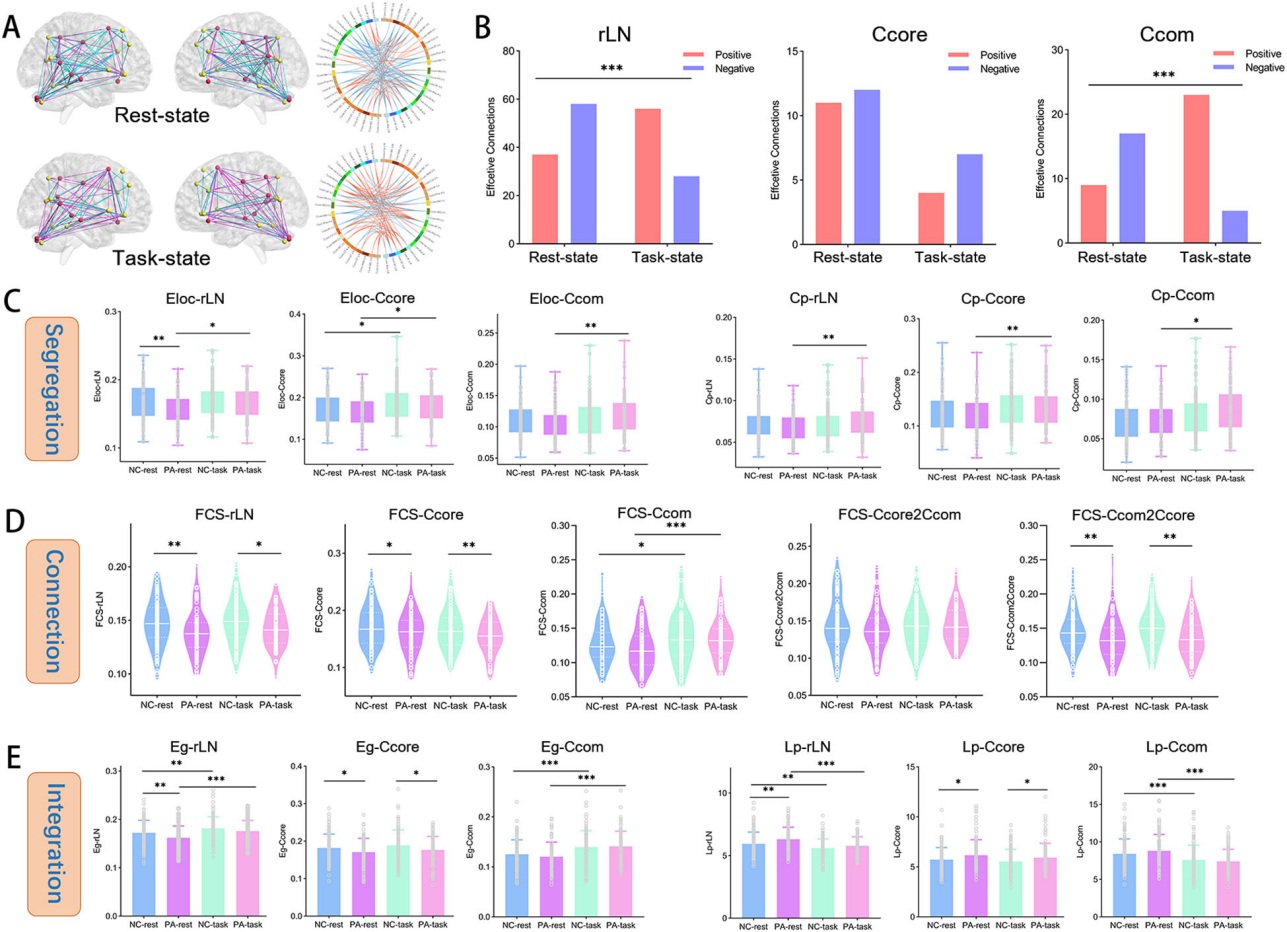
Connectome analyses of the rLN. **(A, B)** Task condition induced a higher proportion of positive effective connections in gliomas compared to controls. **(A)** Significant effective connections in the rLN (free energy, Pp> 95%). Red: positive effective connections; blue: negative effective connections. **(B)** Comparisons in numbers of binary positive and negative effective connections under rest and task conditions. **(C–E)** Comparisons of functional **(C)** segregation of local efficiency (Eloc) and clustering coefficient (Cp), **(D)** connection strength (FCS), and **(E)** integration of global efficiency (Eg) and characteristic path length (Lp) in rest and task conditions. *p < 0.05, **p < 0.01, ***p < 0.001, corrected for multiple comparisons in *post hoc* tests using the Bonferroni–Holm procedure. rLN, reorganized language network; Ccore, core community; Ccom, compensatory community; Ccore2Ccom, connections from core community to compensatory community; Ccom2Ccore, connections from compensatory community to core community.

There were remarkably different binary profiles of effective connections under rest and task conditions (**Supplementary Figure 2**). In brief, compared with controls, a larger proportion of effective connections (61.05%) were negative during the resting state due to the influence of glioma. Conversely, under task conditions, the overwhelming majority of intrinsic connectivity (66.67%) within the rLN were recruited as positive effective connections to tackle the endogenous functional deficiency caused by loss of key hubs (**Figure 7A**). The statistical analyses revealed significant differences in the binary intrinsic connections between patients and controls under resting and task conditions (χ^2^ = 13.72, *p* < 0.001). Further inter-community and intra-community analyses revealed that significantly excitatory effects of task stimuli mainly presented in the intra-connections within Ccom (χ^2^ = 12.61, *p* < 0.001) and the afferent connections from Ccom to Ccore (χ^2^ = 6.17, *p* = 0.021) in patients (**Figure 7B**; **Supplementary Table 6**), which suggested that Ccom played a more proactive role under task conditions.

### Dynamic reconfiguration of the rLN portrayed by topological properties

First, for measures of network segregation (Eloc and Cp), significant within-subject effects of condition were observed at both network and community levels. The between-subject effects of group and interaction effect of condition × group were significant for Eloc [rLN: *F*(1,225) = 6.52, *p* = 0.011; Ccore: *F*(1,225) = 5.51, *p* = 0.02]; and Cp [rLN: *F*(1,225) = 4.05, *p* = 0.045], respectively (**Figure 7C**). Simple-effect analyses revealed that lower Eloc (*p* = 0.007) and Cp (*p* = 0.002) were only present during the resting-state condition, which suggested that the functional segregation was minimally affected by gliomas, and the dysfunctions of the rLN reached a normal onco-functional balance in the task conditions. Second, we found a main effect of within-subject factor of condition for Ccom [*F*(1,225) = 18.66, *p* < 0.001] and between-subject effects of group for rLN, Ccore, and Ccom2Ccore [rLN: *F*(1,225) = 15.52, *p* < 0.001; Ccore: *F*(1,225) = 11.49, *p* = 0.001; Ccom2Ccore: *F*(1,225) = 18.51, *p* < 0.001] in the average FCS of effective connections (**Figure 7D**). Third, for the characters of functional integration (Eg and Lp), the tests for between-subject effect of group revealed significance in rLN [Eg: *F*(1,225) = 10.55, *p* = 0.001; Lp: *F*(1,225) = 9.85, *p* = 0.002] and Ccore [Eg: *F*(1,225) = 9.92, *p* = 0.002; Lp: *F*(1,225) = 9.10, *p* = 0.003]. The simple-effect analyses showed that, for Ccore, significant differences of Eg and Lp existed in both rest and task conditions, but lower Eg and higher Lp were eliminated during task condition in rLN, suggesting that the reorganized onco-functional integration improved to an undifferentiated level comparable to controls under task-induced recruitment of the domain-general community in patients (**Figure 7E**; **Supplementary Table 7**).

### Correlation analyses for onco-functional metaplasticity

For topological indicators, we found that network connection strength (FCS-grade: *R*_100_ = -0.227, *P* = 0.023; FCS-volume: *R*_100_ = -0.207, *P* = 0.039) and network integration [(Eg-grade: *R*_100_ = -0.217, *P* = 0.030; Eg-volume: *R*_100_ = -0.230, *P* = 0.022), not network segregation, displayed significant correlations with pathological features of glioma (grade and volume), which showed that functional integration may have more significant clinical predictive value (**Supplementary Table 8**). Interestingly, the significant associations were only observed at resting state, not for the task condition, and these correlations only exist at the overall connectivity level of tumor-induced rLN, which may be due to the fact that functional connectivity in the resting state constructs the basic framework of the rLN. For behavioral performances, firstly, significant correlations between clinicopathology and language were found for both grade (*R*_100_ = -0.38, *P* < 0.001) and volume (*R*_100_ = -0.20, *P* = 0.046), but not for duration (R_100_ = 0.07, P = 0.474) (**Supplementary Table 9**). Secondly, significant correlations between lateralization and language were found in both the frontal and parietal masks of LI for SN (frontal: *R*_100_ = 0.201, *P* = 0.045; parietal: *R*_100_ = 0.206, *P* = 0.039) and CEN (frontal: *R*_100_ = 0.212, *P* = 0.034; parietal: *R*_100_ = 0.239, *P* = 0.017) activation, not for the temporal mask of SN (*R*_100_ = 0.102, *P* = 0.313) (**Supplementary Table 10**). However, the analyses for activation of 12 significant clusters and language revealed no significant associations between them at the singular cluster level, which again emphasized the synergistic role of functional networks rather than a single brain region in underlying glioma-induced metaplasticity (**Supplementary Table 11**). Thirdly, significant correlations between network properties and language were found in FCS (*R*_100_ = 0.20, *P* = 0.046), Eg (*R*_100_ = 0.22, *P* = 0.028), and Lp (*R*_100_ = -0.27, *P* = 0.007) in the resting state of core language community, not in the task condition (**Supplementary Table 12**).

### Mediation analyses for onco-functional reorganization

**Figure 8:**
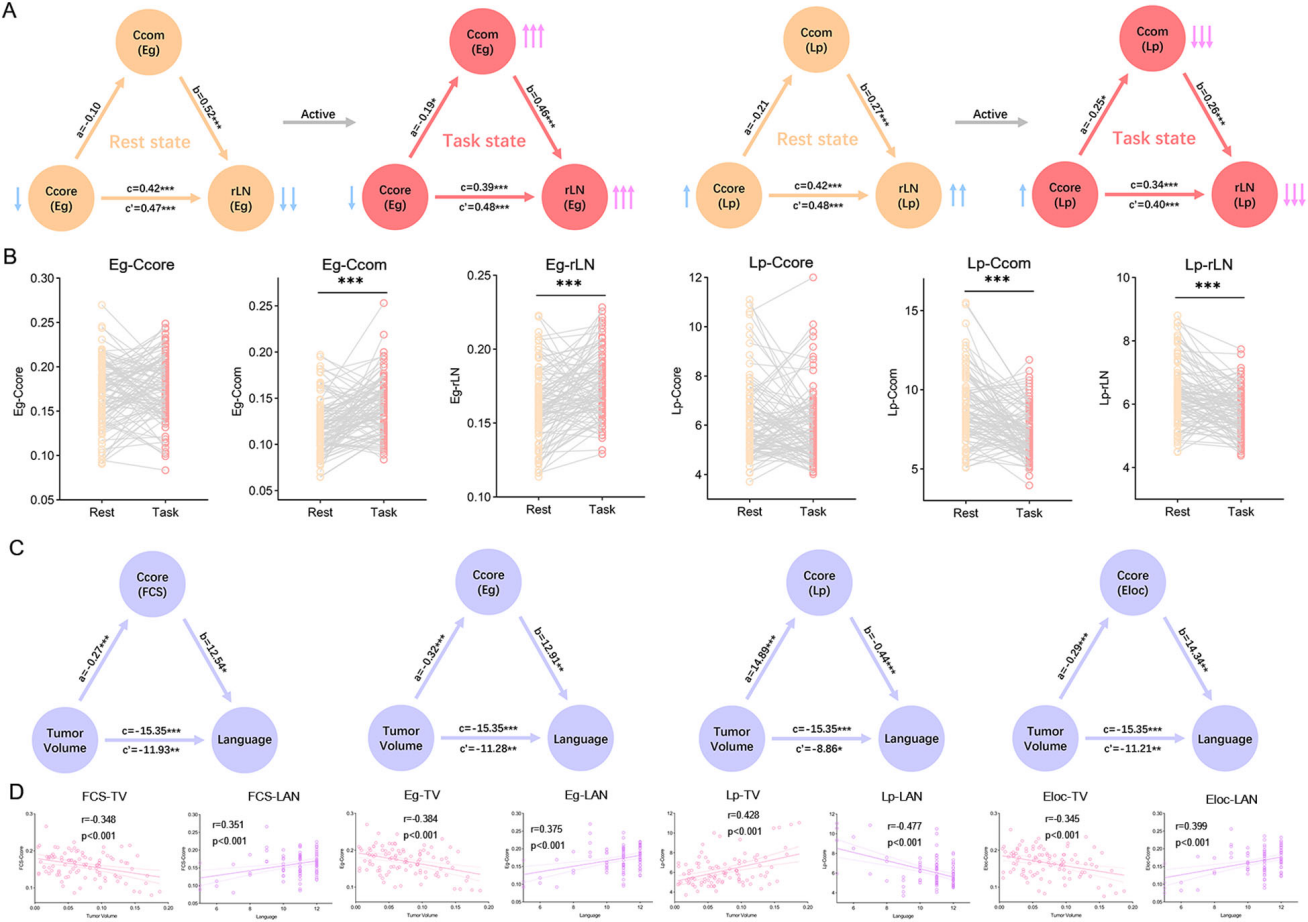
Mediation analyses of compensatory dynamics and clinical relevance. **(A, B)** Neural kinetics underlying the improvement of functional integration (Eg and Lp) induced by recruitment of compensatory community (Ccom) in task activation. **(A)** The mediated effects of Ccom exist in activation (task) but not in baseline (rest) state. **(B)** Pair-wise changes of Eg and Lp in the framework of metaplasticity evoked by task demands. **(C, D)** Mediated pathway from clinicopathology to language behavior via functionally specific core community (FCS, Eg, Lp, and Eloc). *p < 0.05, **p < 0.01, ***p < 0.001.

As shown in **Figures 8A, B** and **Table 5**, only in active condition were the indirect pathways mediated by the Ccom significant (Eg-task: *β* = -0.089, 95% CI = [-0.169, -0.026]; Lp-task: *β* = -0.064, 95% CI = [-0.124, -0.025]). The significant network mediators include FCS, Eg, Lp, and Eloc (**Figures 8C,D**), all of which are restricted to the Ccore under rest condition (FCS: *a* = -0.27, *p* < 0.001; *b* = 12.54, *p* = 0.012; Eg: *a* = -0.32, *p* < 0.001; *b* = 12.91, *p* = 0.008; Lp: *a* = 14.89, *p* < 0.001; *b* = -0.44, *p* < 0.001; Eloc: *a* = -0.29, *p* < 0.001; *b* = 14.34, *p* = 0.002) (**Table 5**). Together with the improved functional integration, we infer that dynamic functional shifts of positive and negative effective connectivity play an essential role in the renormalization of language onco-functional integrity. These results may indicate that glioma-induced language deficits are caused by impairment of the functional integration and segregation of the language-specific community, and rLN is partly resilient to focal lesions because of the dynamic engagement of the domain-general community in a context-dependent manner.

**Table 5:** Mediation analyses for clinical pathological features—the significantly different topological indicators and behavioral language performance.

Mediation factors		Total effect (*c*)			Direct effect (*c*’)			Indirect effect (a × b)		
		*B*	SE	95% CI	*B*	SE	95% CI	*B*	SE	95% CI
Eg	Rest	0.42***	0.05	[0.32, 0.52]	0.47***	0.03	[0.42, 0.53]	-0.05	0.04	[-0.14, 0.03]
Eg	Task	0.39***	0.05	[0.30, 0.49]	0.48***	0.03	[0.42, 0.55]	-0.09*	0.04	[-0.17, -0.03]
Lp	Rest	0.42***	0.05	[0.33, 0.52]	0.48***	0.03	[0.43, 0.54]	-0.06	0.03	[-0.12, 0.002]
Lp	Task	0.34***	0.04	[0.26, 0.41]	0.40***	0.03	[0.35, 0.45]	-0.06*	0.03	[-0.12, -0.03]
FCS	Rest	-15.35***	3.71	[-22.71, -7.99]	-11.93**	3.85	[-19.57, -4.29]	-3.42*	1.93	[-7.59, -0.14]
Eg	Rest	-15.35***	3.71	[-22.71, -7.99]	-11.28**	3.89	[-19.00, -3.56]	-4.07*	2.14	[-8.74, -0.32]
Lp	Rest	-15.35***	3.71	[-22.71, -7.99]	-8.86*	3.83	[-16.45, -1.27]	-6.49*	3.07	[-13.00, -1.18]
Eloc	Rest	-15.35***	3.71	[-22.71, -7.99]	-11.21**	3.78	[-18.72, -3.70]	-4.14*	2.15	[-8.80, -0.50]

## Discussion

This study focused on patterns of language reorganization caused by glioma using a combination of resting-state and naming-task fMRI data, which constitutes an integrated approach combining spDCM analyses and connectome methods to study the functional interactions within the rLN. Our goal was to assess whether the clinicopathological factors are associated with preferred increased activation clusters in the perilesional and remote brain areas and to further explore the dynamic interactions among subsystems of the rLN from rest to task states. First, we found that clusters with increased activation, potentially reflecting compensatory recruitment, were widely distributed within domain-general networks, exhibiting greater right-hemispheric predominance on lateralization analysis. Second, altered activation showed associations with tumor location and grade, which may reflect metaplasticity-related reorganization patterns associated with clinicopathologic features of glioma. Third, compared with controls, task-related computational complexity was associated with a greater number of positive effective connections across rLN and the subnetwork of Ccom. Fourth, Ccom may operate by improving the onco-functional balance between integration and segregation to reconfigure the linguistic information transmission during task conditions. Fifth, mediation analyses suggested direct and indirect associations among clinicopathological factors, network topologies, and language performance. Taken together, our findings suggest that domain-general networks may contribute to glioma-induced language network reorganization at both the cluster and connectome levels, potentially reflecting compensatory processes. In this study, we observed widely distributed cortical areas with increased activation in patients relative to controls, similar to perilesional or contralateral homologous plasticity observed in damaged brain areas during recovery from acute illnesses (e.g., stroke or trauma) (55–57). Our findings suggest a spatial dissociation between the distribution of increased activation clusters and tumor location. Specifically, larger tumor volumes were associated with decreased perilesional activation and increased remote activation, which may reflect the disruptive effects of tumor expansion on perilesional tissue integrity and a corresponding shift in neural recruitment patterns. When we inspect the spatial distribution of the increased activation clusters at the functional network level, we observed a mainly overlapping location within areas of domain-general networks, especially the cingulo-opercular and frontoparietal networks. These networks have been linked to the initiation and maintenance of cognitive control for goal-directed behaviors, suggesting that different task demands may be associated with dynamic reconfiguration of functional connectivity within rLN (58–61). More recently, studies have regarded linguistic functions as arising from flexible configurations between domain-general and language-specific networks, which are modulated by exogenous linguistic complexity or endogenous functional integrity (13, 62, 63). Liang et al. reported that greater cognitive effort induced higher-level connections in lateral frontoparietal cortices of the executive control network, which was colocalized with the “classic” brain areas associated with language production and comprehension (64). Studies about the role of domain-general networks after stroke also suggested that the activity in the right inferior frontal gyrus was correlated with the upregulation of working memory or executive control for recovery from post-stroke aphasia (65–68). These results are consistent with our observation of predominantly right-hemispheric activation during naming tasks in patients, which may reflect compensatory recruitment. The increased activation clusters are mainly assigned to overlapping areas of the domain-general and language-specific networks, which also reflect an increase in cognitive communication and cooperation to reconfigure linguistic processing deficiencies under gliomas.

Although we cannot decompose specific linguistic processing (such as phonology, morphology, syntax, and semantics) from the assumed compensatory clusters with greater activation in naming task fMRI sets (69–71), we do find some functional brain areas directly associated with distinct glioma clinicopathological factors, with potential applications for network neurosurgery—that is, right hemispheric gliomas were associated with higher functional activation in the right anterior insula, the pars triangularis of the left inferior frontal gyrus, and the left precuneus gyrus, irrespective of tumor grade and lobe. Similarly, frontal lobe gliomas were associated with higher lobe-specific activations in the right postcentral gyrus and inferior temporal gyrus. The low-grade gliomas are independently associated with higher activations in the left anterior insula, right fusiform, and right lingual gyrus, but with lower activation in the left angular gyrus. These findings are consistent with previous results induced by gliomas, which showed that tumor location and grade were independent factors affecting language processing and linguistic functions (72–74). As many studies have demonstrated, the neural mechanisms contributing to language reorganization do not merely depend on localized activation (75, 76); however, the interactions between language and other cognitive systems may provide onco-functional maps relevant to the clinicopathology of gliomas, which may indicate the potential treatment targets for postoperative brain stimulation across patient populations who share common attributes (6, 41, 77, 78). Furthermore, the therapeutic effects of analogous brain regions with greater activation have been confirmed to accelerate language recovery in poststroke aphasia (79, 80).

Additionally, we also studied perilesional and remote neuroplasticity for glioma-induced greater activations using VLSM analyses, which showed reverse correlations between perilesional and remote activation with tumor volume. Specifically, as the tumor size increased, the activity of the perilesional clusters was gradually decreased; instead, remote compensation became more active. The more active participation of the remote increased activation cluster is consistent with reports from previous aphasia treatment studies, which concluded that the increased activation of lesion-remote areas was associated with improved language performance after aphasia treatment (66, 81, 82). Our findings may offer an alternative perspective on the “disinhibition” hypothesis, which posits a conflicting view that the loss of transcallosal inhibition to contralateral homotopic cortex hinders recovery. That might be a hypothesis that perilesional and remote compensation are integral parts of the compensatory network. When perilesional compensation gradually becomes inefficient due to the destructive effects of tumor invasion and compression, the phenomenon of compensatory metastasis occurs and the remote clusters are flexibly recruited to support the compensatory capacities (2, 3, 6).

After completing the analyses of compensatory associations with clinicopathological factors at the cluster level, we also provided the topological analyses at the network and community levels to follow the latest concepts of metaplasticity and network neurosurgery. Importantly, we found that the glioma-induced rLN showed an onco-functional reconfiguration modulated by task demands when compared with the resting state with task-based network properties. Positive effective connections within Ccom, as well as the afferent effective connections from Ccom to Ccore of the rLN, were more frequently observed during naming task conditions, which may reflect the increased engagement of domain-general network components, potentially acting as an excitatory-rich club to modulate linguistic processing demands when gliomas affect core language network hubs (83). In agreement with this concept, some meta-analyses have reframed the organization of the language system as a hierarchical architecture consisting of functionally specialized “core” and domain-general “periphery” components, the dynamic interactions among which are modulated by extrinsic linguistic complexity or intrinsic functional capability (63, 84, 85). This may suggest that, to some extent, increased external computational demands in healthy subjects may be comparable to impaired or decreased internal functional capacities in glioma patients, as domain-general resources need to be more actively recruited to support the inadequate processing demands of the language-specific Ccore.

Next, we asked what kinds of topological properties of the rLN are improved by the flexible engagement of Ccom for glioma patients. The framework of graphical matrices based on intrinsic connectivity offers a useful approach for characterizing glioma-induced metaplasticity, as it may capture aspects of network robustness, adaptation, and evolution that extend beyond the indirectly measured BOLD signal (86, 87). By assessing state-wise topological characteristics, our findings suggest that the dynamics of onco-functional equilibrium are organized in a task-dependent and context-sensitive manner (49, 50). Specifically, reduced network integration and segregation, with lower global/local efficiency and higher characteristic short path length at rest, returned to normal functional balance with the flexible engagement of domain-general components under task conditions. The preserved functional segregation, as indexed by the Cp at both rest and task conditions, is consistent with the robustness and resilience of the rLN to tumor-related effects, reflecting attributes similar to those of a small-world network. Additionally, we observed reduced FCS within rLN- and language-specific Ccore, but not within Ccom under both rest and task conditions. The impaired functional connectivity remains remarkable even with the compensatory engagement, which may partially explain the behavioral deficits in a portion of glioma patients (88–90). Recent studies revealed that the domain-separation language network dynamics in resting and task states share a common scaffold or backbone of intrinsic effective connectivity that supports flexible functional segregation and integration during language and speech processing (37, 50, 91–93). Thus, these network-level changes, characterized by increased activation clusters (Ccom) embedding into language-specific Ccore, may reflect a reorganized patterns of domain-general systems participating in language metaplasticity through a task-dependent manner. Pairwise associations among the tumor pathological factors, network functional characteristics, and clinical language behaviors have been widely reported in glioma patients (89, 94, 95); however, the mediating pathways underlying the clinicopathological factors to clinical language behaviors still seem unclear. Here we identified a potential network-based neuropathological pathway linking tumor volume to language performance, suggesting that gliomas located in language areas may impair language performance partially mediated by abnormal functional strength and the efficiency of language-specific Ccore network or core community defined by this study. Although the mediating pathways are useful for developing directional concepts of network neurosurgery, they do not imply that the functional topologies of language-specific subnetwork represent simple relayed inferences affecting language behavior in glioma patients. It remains possible that the Ccom may be involved in neural circuits as antagonistic effects, given the cerebral plasticity phenomena (34, 96). Thus, interactions among tumor factors, network characteristics, and language performance should be considered to incorporate the dynamic potential of functional reorganization in operative planning (97)—for example, recent studies have demonstrated that functional integration between glioblastoma and normal brain tissue negatively affects both patient survival and performance on language tasks (98–100). Further studies should verify the specific role of these normal regions for compensatory mapping and investigate their functional variability in language recovery after glioma resection (101–103). The relationship between these connectome characteristics and language may have clinical value for predicting behavioral manifestations in the era of network neurosurgery.

The concept of network neurosurgery represents a clinical translation of network neuroscience, emphasizing glioma-induced remodeling of network connectivity and critical nodes (104). For neurosurgical practice, this theoretical framework entails a fundamental evolution in therapeutic paradigm: rather than merely asking whether a tumor invades a canonical eloquent area (e.g., Broca’s and Wernicke’s regions), this approach evaluates whether resection would disrupt a network configuration that the brain currently relies upon for language performance and whether remaining network resources possess sufficient plasticity to maintain function after nodal disruption (105). This paradigm shift is characterized by three key aspects, namely (1), it emphasizes the importance of neural circuit key nodes, expanding onco-functional research from localized lesion sites to brain network through integrating structural and functional connectomics (2); neurosurgeons can identify critical network hubs via topological analysis and whole-brain modeling, which may enable precise surgical approach selection and trajectory planning to minimize disruption to functional network; and (3) functional preservation has evolved from tumor-centric to network-oriented strategies, thereby enhancing postoperative rehabilitation and predicting patient prognosis. By leveraging the established neuroplasticity in low-grade gliomas, staged surgical strategies can promote subcortical pathway remodeling, enabling safer maximal resection without postoperative language deficits (3, 106). However, significant challenges remain regarding the standardization of language network construction protocols, definition of linguistic key nodes, reliability of the rLN intervention theory, and reproducibility of individualized language network analyses (106, 107). The group-level findings presented in this study regarding language network topological alterations and their associations with glioma clinicopathological features may provide a modeling framework to conduct subsequent individualized language network assessments. Understanding the cross-talk within the rLN, especially how functional frameworks of integration and segregation affect behavioral outcomes, may therefore inform preoperative patient counseling regarding postoperative risks and assist with preoperative surgical planning. Undoubtedly, driven by advances in artificial intelligence, big data functional modeling, and minimally invasive techniques, the trajectory toward a new era of individualized network neurosurgery will remain steadfast (16).

### Limitations

A limitation of this study is the activity increases observed in patients relative to controls based on BOLD signal magnitude that may reflect compensatory processes, but these changes are a phenomenological description rather than a definitive proof of functional efficacy. Although they are often hypothesized to support performance, such activation changes do not guarantee behavioral benefit and may sometimes reflect maladaptive plasticity. Furthermore, as a group-level observational study, our findings represent averaged trends. The ICA, ANOVA, and VLSM results should be interpreted as identifying reliable group-specific patterns within this cohort rather than as individual, universal maps. These results should be interpreted as generating hypotheses for future longitudinal and individualized investigations rather than as definitive evidence for direct clinical application. Another limitation of this study is the simplification of clinicopathological variables into binary categories. While this approach facilitated robust group-level statistical analysis, it inherently reduces anatomical and biological granularity—for instance, the “frontal” category encompasses diverse functional sub-regions, and “high grade” includes various molecular profiles. Caution is warranted when extrapolating these findings to individualized clinical applications, as the binary model may overlook critical inter-individual variability. Additionally, the cross-sectional design of this study precludes the inference of causal or longitudinal relationships between glioma progression and language network reorganization. The observed associations between clinicopathological factors and network topologies reflect a single time-point snapshot, which cannot capture the temporal dynamics of compensatory mechanisms or distinguish between cause and consequence. Finally, volume measurements were derived from non-contrast T1 images and are therefore less precise than contrast-enhanced volumetric assessments, and the binary grade classification employed here represents a deliberate simplification for group-level statistical analysis and does not reflect contemporary clinical standards, which prioritize integrated molecular markers (e.g., IDH mutation status, 1p/19q codeletion, and MGMT promoter methylation) over histological grade alone. Future work combining cerebrovascular reactivity mapping (e.g., breath-hold fMRI) and subregional tumor segmentation (contrast-enhancing/necrotic/edema compartments) could better disentangle neurovascular uncoupling heterogeneity and improve result robustness.

## Conclusions

This study provides insights into the potential mechanisms of onco-functional reorganization underlying presurgical language network alterations across both rest and task states. The observed flexible coordination between language-specific and domain-general networks may reflect onco-functional reallocation patterns associated with glioma progression. Furthermore, our mediation analyses suggest that topological frameworks of functional integration and segregation may be associated with the dynamic aspects of language network reorganization. These preoperative neuroplastic patterns may inform future efforts to identify patient subgroups with varying potential for functional rehabilitation, contributing to the development of personalized therapeutic strategies in the emerging field of network neurosurgery.

## Data Availability

The datasets presented in this study can be found in online repositories. The names of the repository/repositories and accession number(s) can be found in the article/**Supplementary Material**.

## Supplementary material

Supplementary Figure 1 Locations of nodes for the rLN, which is composed of the core community (red, Ccore) of the language-specific network identified by ICA and compensatory community (blue, Ccom) detected by activation test.

Supplementary Figure 2 Commonalities of effective connections in controls and patients under rest and task states (free energy, *p* > 95%).

Supplementary Table 1 MNI coordinates of clusters in SN identified by ICA (*p* < 0.05, FWE-corrected).

Supplementary Table 2 MNI coordinates of clusters in CEN identified by ICA (*p* < 0.05, FWE-corrected).

Supplementary Table 3 MNI coordinates of clusters in LN identified by ICA (*p* < 0.05, FWE-corrected).

Supplementary Table 4 Lateralization comparison between patients and controls in laterality index.

Supplementary Table 5 Numbers of binarized LI statistical analyses for masks of SN, CEN, and LN.

Supplementary Table 6 Comparison of effective connectivity between patients and controls under rest and task conditions.

Supplementary Table 7 Comparisons on topological alterations between gliomas and controls under rest and task conditions.

Supplementary Table 8 Correlations between clinicopathological factors and significantly different topological indicators (PA vs. NC, *p* < 0.05) across patients.

Supplementary Table 9 Correlations between clinicopathological factors and behavioral performance across patients.

Supplementary Table 10 Correlations between significantly different LI indicators (PA vs. NC, *p* < 0.05) and behavioral performance across patients.

Supplementary Table 11 Correlations between significantly compensatory activation (PA vs. NC, FDR-corrected, *p* < 0.05) and behavioral performance across patients.

Supplementary Table 12 Correlations between the significantly topological indicators (PA vs. NC) and behavioral performance across patients.
