# Supplementary material for: The onco-functional reorganization of language network underlying metaplasticity induced by gliomas
Source: Front Oncol. 2026 May 29;16:1850713. doi: 10.3389/fonc.2026.1850713 (PMC13259869; doi:10.3389/fonc.2026.1850713)
Supplement: Supplementary Figure 1 — Locations of nodes for the rLN, which is composed of the core community (red, Ccore) of the language-specific network identified by ICA and compensatory community (blue, Ccom) detected by activation test. [file DataSheet1.docx]

**Functional connection strength**

For a network, the strength is the sum of the edge weights linking all connected nodes. The average strength is the mean of the sum strength in the network. The formula of mean FCS is expressed as:

$$S_{p}\left( G \right)=\frac{1}{N}\sum_{i\in G} S(i),$$

where $S\left( i \right)$is the sum of the edge weight linking to node $i$. $S_{p}\left( G \right)$ is the average of the sum strength across the network.

**Functional segregation**

Functional segregation refers to the specialized processing ability among densely connected modules in a network. Measures of functional segregation are commonly based on the clustering coefficient (Cp) and local efficiency (Eloc). The average Cp quantifies the number of connections among the direct neighbors of a node as a proportion of the maximum number of possible connections. If a node’s neighbors are densely interconnected, they form a cluster that shares specialized information(1). The weighted Cp of a node is expressed as follows:

$$C^{w}\left( i \right)=\frac{2}{k_{i}(k_{i}-1)}\sum_{j,k} {(\bar{w}_{ij}\bar{w}_{jk}\bar{w}_{ki})}^{1/3},$$

where $k_{i}$ is the degree of node $i$, and $\bar{w}$ is the weight, which is scaled by the mean of all weights to control each participant’s cost at the same level.

The local efficiency reflects how well the information is transferred within the neighbors of a given node(2). The Eloc of a network is defined as the average of local efficiencies of each node, that is:

$$E_{loc}\left( G \right)=\frac{1}{N}\sum_{i\in G} E_{glob}(G_{i}),$$

where $G_{i}$ denotes the subgraph composed of the nearest neighbors of node $i$.

**Functional integration**

Functional integration relates to the capacity of a network rapidly combining associated information from distributed brain regions. Measures of functional integration include the characteristic path length (Lp) and global efficiency (Eg). Lp is a global metric that describes the average shortest path length across all possible pair of nodes. For weighted networks, the length of each weighted edge was assigned by computing the reciprocal of the edge weight(3). The weighted $L_{p}^{w}$ of a network is computed as follows, where $N$ is the number of nodes in the network, the $L_{p}^{w}$ is the ability for information propagation in parallel, that is:

$$L_{p}^{w}=\frac{1}{N\left( N-1 \right)}\sum_{i=1}^{N} \sum_{j\neq i}^{N} \frac{1}{L^{w}(i,j)} ,$$

The global efficiency is associated with how well a network supports parallel information transfer between nodes via multiple series of edges. The path length between node i and node j is defined as the sum of the edge lengths along the path, and $L_{ij}$ is the shortest path length between node $i$ and $j$(4). $E_{g}\left( G \right)$ can be computed as follows:

$$E_{g}\left( G \right)=\frac{1}{N(N-1)}\sum_{i\neq j\in G} \frac{1}{L_{ij}},$$

**Refference:**

1. Watts DJ, Strogatz SH. Collective Dynamics of 'Small-World' Networks. *Nature* (1998) 393(6684):440-2. Epub 1998/06/12. doi: 10.1038/30918.

2. Latora V, Marchiori M. Efficient Behavior of Small-World Networks. *Physical review letters* (2001) 87(19):198701. Epub 2001/11/03. doi: 10.1103/PhysRevLett.87.198701.

3. Telesford QK, Joyce KE, Hayasaka S, Burdette JH, Laurienti PJ. The Ubiquity of Small-World Networks. *Brain connectivity* (2011) 1(5):367-75. Epub 2011/01/01. doi: 10.1089/brain.2011.0038.

4. Vragović I, Louis E, Díaz-Guilera A. Efficiency of Informational Transfer in Regular and Complex Networks. *Physical review E, Statistical, nonlinear, and soft matter physics* (2005) 71(3 Pt 2A):036122. Epub 2005/05/21. doi: 10.1103/PhysRevE.71.036122.


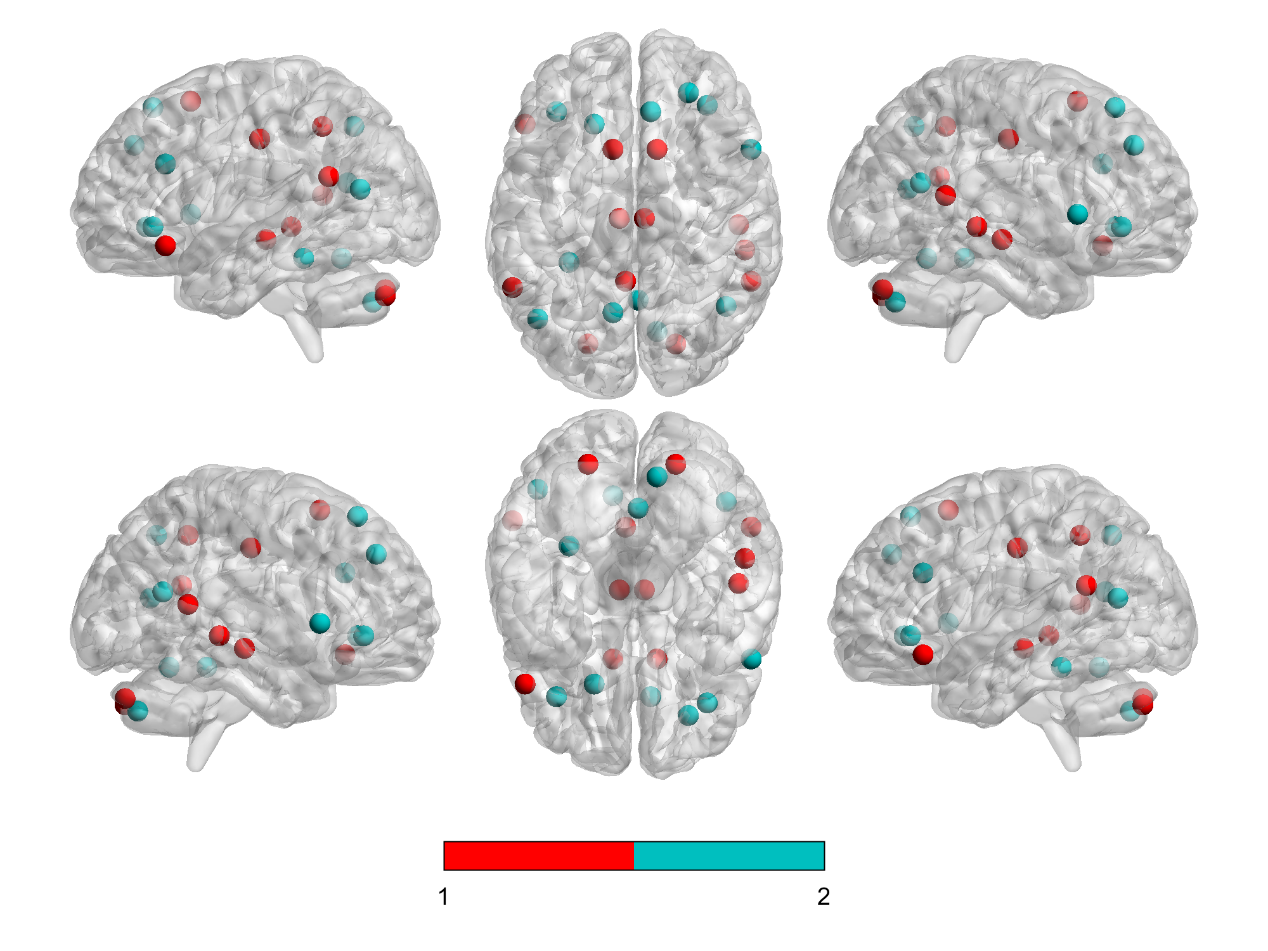
**Figure S1.** The locations of nodes for rLN, which is composed of core community (red, Ccore) of language-specific network identified by ICA and compensatory community (blue, Ccom) detected by activation test.

rLN=reorganized language network, Ccore=core community, Ccom=compensatory community.


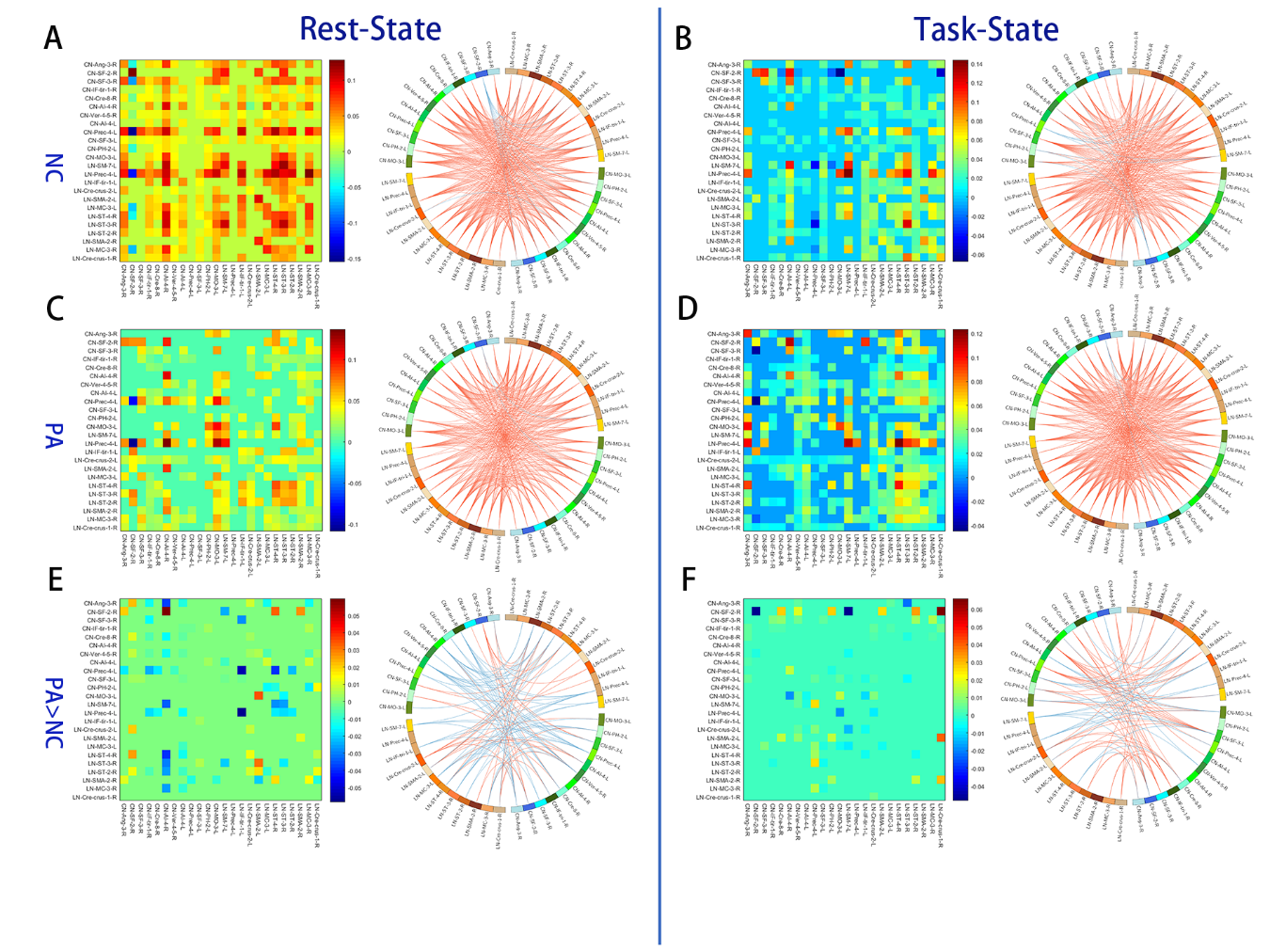
**Figure S2.** Commonalities and differences of effective connections in controls and patients under rest and task states (free energy, p>95%).

Red lines indicate positive intrinsic connectivity, bule lines represent negative intrinsic connectivity in the Circos circles. NC=normal control, PA= glioma patients.

**Table S1.** The MNI coordinates of clusters in SN identified by ICA (p<0.05, FWE-corrected).

| Regions  (AICHA) | Sides | Peak coordinates (MNI) | Statistics | | | |
| --- | --- | --- | --- | --- | --- | --- |
|  |  |  | P_FWE-coor_ | T | P_uncoor_ | k |
| Insula-anterior-4 | Left | -39 21 -6 | <0.001 | 60.02 | <0.001 | 3305 |
| Temporal-Mid-1 | Left | -54 -18 -15 | <0.001 | 11.62 | <0.001 | subcluster |
| Temporal-Mid-3 | Left | -54 -33 -9 | <0.001 | 11.29 | <0.001 | subcluster |
| Supp-Motor-Area-3 | Left | -6 15 51 | <0.001 | 16.35 | <0.001 | 798 |
| Cingulate-1 | Left | -6 24 27 | <0.001 | 10.98 | <0.001 | subcluster |
| Frontal-Sup-Medial-2 | Left | -6 45 27 | <0.001 | 10.11 | <0.001 | subcluster |
| Sup-Temporal-4 | Left | -57 -48 24 | <0.001 | 15.39 | <0.001 | 301 |
| Cuneus-1 | Left | 0 -84 33 | <0.001 | 13.18 | <0.001 | 681 |
| Precuneus-1 | Left | -15 -57 12 | <0.001 | 12.40 | <0.001 | subcluster |
| Caudate-6 | Left | -12 -9 21 | <0.001 | 12.10 | <0.001 | subcluster |
| Cerebellum-8 | Left | -18 -60 -48 | <0.001 | 8.19 | <0.001 | 61 |
| Insula-anterior-3 | Right | 39 27 -9 | <0.001 | 45.73 | <0.001 | 2232 |
| Insula-anterior-4 | Right | 42 12 -3 | <0.001 | 39.98 | <0.001 | subcluster |
| Inf-Frontal-2 | Right | 51 36 24 | <0.001 | 8.02 | <0.001 | subcluster |
| Frontal-Sup-Medial-3 | Right | 0 18 48 | <0.001 | 14.75 | <0.001 | 448 |
| Cingulum-Ant-1 | Right | 0 33 -3 | <0.001 | 10.05 | <0.001 | subcluster |
| Cingulum-Ant-2 | Right | 6 30 21 | <0.001 | 7.66 | <0.001 | subcluster |
| Cuneus-1 | Right | 3 -84 33 | <0.001 | 13.25 | <0.001 | 1427 |
| Caudate-7 | Right | 12 -6 21 | <0.001 | 13.12 | <0.001 | subcluster |
| Parietooccipital-4 | Right | 27 -39 12 | <0.001 | 12.03 | <0.001 | subcluster |
| Sup-Temporal-3 | Right | 51 -30 -6 | <0.001 | 10.49 | <0.001 | 144 |
| Sup-Temporal-2 | Right | 54 -18 -15 | <0.001 | 7.90 | <0.001 | subcluster |
| ParaHippocampal-5 | Right | 12 -33 -18 | <0.001 | 8.68 | <0.001 | 62 |
| SupraMarginal-5 | Right | 60 -42 30 | <0.001 | 8.18 | <0.001 | 85 |

**Table S2.** The MNI coordinates of clusters in CEN identified by ICA (p<0.05, FWE-corrected).

| Regions  (AICHA) | Sides  (L/R) | Peak coordinates (MNI) | Statistics | | | |
| --- | --- | --- | --- | --- | --- | --- |
|  |  |  | P_FWE-coor_ | T | P_uncoor_ | k |
| Precuneus-1 | Left | -9 -57 12 | <0.001 | 49.74 | <0.001 | 3131 |
| Occipital-Mid-4 | Left | -39 -75 24 | <0.001 | 46.34 | <0.001 | subcluster |
| ParaHippocampal-5 | Left | -30 -36 -18 | <0.001 | 31.73 | <0.001 | subcluster |
| Sup-Frontal-5 | Left | -24 12 48 | <0.001 | 38.46 | <0.001 | 3419 |
| Sup-Frontal-4 | Left | -24 27 39 | <0.001 | 33.91 | <0.001 | subcluster |
| Frontal-Med-Orb-2 | Left | 0 51 -15 | <0.001 | 17.75 | <0.001 | subcluster |
| Cerebellum-9 | Left | -9 -51 -48 | <0.001 | 16.24 | <0.001 | 173 |
| Fusiform-7 | Left | -15 -87 -6 | <0.001 | 10.79 | <0.001 | 374 |
| Cerebellum-Crus-1 | Left | -15 -72 -33 | <0.001 | 10.40 | <0.001 | subcluster |
| Cerebellum-Crus-2 | Left | -12 -84 -39 | <0.001 | 9.41 | <0.001 | subcluster |
| Temporal-Mid-1 | Left | -60 -6 -24 | <0.001 | 9.80 | <0.001 | 80 |
| Angular-3 | Right | 45 -63 21 | <0.001 | 59.77 | <0.001 | 9567 |
| Precuneus-1 | Right | 12 -54 12 | <0.001 | 56.12 | <0.001 | subcluster |
| Sup-Frontal-5 | Right | 24 30 39 | <0.001 | 38.88 | <0.001 | subcluster |

**Table S3.** The MNI coordinates of clusters in LN identified by ICA (p<0.05, FWE-corrected).

| Regions  (AICHA) | Sides | Peak coordinates (MNI) | Statistics | | | |
| --- | --- | --- | --- | --- | --- | --- |
|  |  |  | P_FWE-coor_ | T | P_uncoor_ | k |
| SupraMarginal-7 | Left | -57 -51 21 | <0.001 | 53.80 | <0.001 | 6004 |
| Precuneus-4 | Left | -3 -48 45 | <0.001 | 29.43 | <0.001 | subcluster |
| Frontal-Inf-Tri-1 | Left | -51 27 -12 | <0.001 | 25.05 | <0.001 | subcluster |
| Cerebellum-Crus-2 | Left | -21 -78 -36 | <0.001 | 21.86 | <0.001 | 403 |
| Supp-Motor-Area-2 | Left | -9 15 57 | <0.001 | 19.84 | <0.001 | 305 |
| Putamen-3 | Left | -12 0 3 | <0.001 | 12.92 | <0.001 | 612 |
| Thalamus-9 | Left | 0 -24 -6 | <0.001 | 12.08 | <0.001 | subcluster |
| Caudate-3 | Left | -15 24 -9 | <0.001 | 8.72 | <0.001 | subcluster |
| Cingulum-Mid-3 | Left | -6 -18 39 | <0.001 | 11.35 | <0.001 | 94 |
| Cerebellum-9 | Left | -6 -42 -57 | <0.001 | 8.16 | <0.001 | 135 |
| Cerebellum-9 | Left | -9 -51 -48 | <0.001 | 7.49 | <0.001 | subcluster |
| Sup-Temporal-4 | Right | 57 -48 12 | <0.001 | 66.50 | <0.001 | 110960 |
| Sup-Temporal-3 | Right | 54 -33 -3 | <0.001 | 50.32 | <0.001 | subcluster |
| Sup-Temporal-2 | Right | 51 -21 -9 | <0.001 | 48.26 | <0.001 | subcluster |
| Supp-Motor-Area-2 | Right | 12 15 57 | <0.001 | 22.64 | <0.001 | 372 |
| Cingulum-Mid-3 | Right | 6 -18 39 | <0.001 | 12.35 | <0.001 | 118 |
| Cerebellum-Crus-1 | Right | 21 -78 -33 | <0.001 | 21.57 | <0.001 | 278 |

**Table S4.** Lateralization comparison between patients and controls in laterality index.

| Mask | Mean±SD | | T | P |
| --- | --- | --- | --- | --- |
|  | NC | PA |  |  |
| SN | -0.26±0.27 | -0.23±0.32 | 0.55 | 0.58 |
| SN-F | 0.22±0.32 | 0.10±0.37 | 2.79 | **0.006** |
| SN-P | 0.16±0.38 | -0.07±0.41 | 4.24 | **<0.001** |
| SN-T | 0.09±0.34 | -0.05±0.41 | 2.86 | **0.005** |
| CEN | -0.04±0.24 | -0.07±0.26 | 0.74 | 0.46 |
| CEN-F | 0.27±0.33 | 0.12±0.42 | 2.95 | **0.004** |
| CEN-P | 0.17±0.33 | 0.05±0.34 | 2.71 | **0.007** |
| CEN-T | -0.05±0.29 | -0.09±0.34 | 0.87 | 0.39 |
| LN | -0.31±0.24 | -0.28±0.25 | 1.14 | 0.26 |
| LN-F | 0.16±0.37 | 0.10±0.38 | 1.18 | 0.24 |
| LN-P | 0.01±0.38 | -0.04±0.39 | 0.85 | 0.40 |
| LN-T | -0.31±0.29 | -0.37±0.30 | 1.64 | 0.10 |

CEN=central executive network, SN=salience network, LN=language network; NC=normal control, PA=glioma patients, F=frontal, P=parietal, T=temporal.

| Mask | Left(NC/PA) | Binary(NC/PA) | Right(NC/PA) | χ2 | P |
| --- | --- | --- | --- | --- | --- |
| SN | 9/12 | 38/32 | 80/56 | 2.00 | 0.367 |
| SN-F | 75/46 | 38/31 | 14/23 | 6.73 | **0.035** |
| SN-P | 65/31 | 31/24 | 25/45 | 16.80 | **<0.001** |
| SN-T | 49/26 | 52/38 | 26/36 | 7.74 | **0.021** |
| CEN | 20/14 | 71/57 | 36/29 | 0.13 | 0.935 |
| CEN-F | 74/48 | 40/29 | 13/36 | 17.33 | **<0.001** |
| CEN-P | 58/39 | 49/29 | 20/32 | 8.53 | **0.014** |
| CEN-T | 23/21 | 65/40 | 39/39 | 2.87 | 0.238 |
| LN | 5/5 | 27/29 | 95/66 | 2.11 | 0.347 |
| LN-F | 65/41 | 36/38 | 26/31 | 2.85 | 0.241 |
| LN-P | 41/32 | 47/32 | 39/36 | 0.879 | 0.645 |
| LN-T | 10/4 | 27/21 | 90/75 | 1.50 | 0.474 |

**Table S5.** Numbers of Binarized LI statistical analyses for masks of SN, CEN and LN.

CEN=central executive network, SN=salience network, LN=language network; NC=normal control, PA=glioma patients, F=frontal, P=parietal, T=temporal.

**Table S6.**  Comparison of effective connectivity between patients and controls under rest and task conditions.

| Group | Effective connectivity | | χ2 | P Value |
| --- | --- | --- | --- | --- |
|  | Positive | Negative |  |  |
| **rLN** |  |  |  |  |
| Rest | 37 | 58 | 13.72 | **<0.001** |
| Task | 56 | 28 |  |  |
| **Ccore** |  |  |  |  |
| Rest | 11 | 12 | 0.40 | 0.529 |
| Task | 4 | 7 |  |  |
| **Ccom** |  |  |  |  |
| Rest | 9 | 17 | 12.61 | **<0.001** |
| Task | 23 | 5 |  |  |
| **Ccore2Ccom** |  |  |  |  |
| Rest | 11 | 14 | 2.17 | 0.141 |
| Task | 15 | 8 |  |  |
| **Ccom2Ccore** |  |  |  |  |
| Rest | 6 | 15 | 5.31 | **0.021** |
| Task | 14 | 8 |  |  |

rLN=reorganized language network, Ccore=core community, Ccom=compensatory community, Ccore2Ccom=connections from core community to compensatory community, Ccom2Ccore= connections from compensatory community to core community.

**Table S7.** Comparisons on topological alterations between gliomas and controls under rest and task conditions.

| Topological characters | Within-subject effect  (Condition) | | Between-subject effect  (Group) | | Interactive effect  (Condition x Group) | |
| --- | --- | --- | --- | --- | --- | --- |
|  | F | p | F | p | F | p |
| **Eloc** |  |  |  |  |  |  |
| rLN | 6.12 | **0.014** | 6.52 | **0.011** | 1.87 | 0.173 |
| Ccore | 10.25 | **0.002** | 5.51 | **0.020** | 0.01 | 0.963 |
| Ccom | 10.96 | **0.001** | 0.01 | 0.937 | 3.24 | 0.073 |
| **Cp** |  |  |  |  |  |  |
| rLN | 7.69 | **0.006** | 0.01 | 0.950 | 4.05 | **0.045** |
| Ccore | 14.90 | **<0.001** | 0.36 | 0.550 | 1.80 | 0.181 |
| Ccom | 19.72 | **<0.001** | 2.24 | 0.136 | 2.72 | 0.101 |
| **FCS** |  |  |  |  |  |  |
| rLN | 2.64 | 0.105 | 15.52 | **<0.001** | 0.82 | 0.367 |
| Ccore | 1.97 | 0.162 | 11.49 | **0.001** | 0.08 | 0.784 |
| Ccom | 18.66 | **<0.001** | 1.54 | 0.216 | 1.67 | 0.197 |
| Ccore2Ccom | 0.93 | 0.336 | 0.57 | 0.452 | 2.30 | 0.131 |
| Ccom2Ccore | 1.73 | 0.189 | 18.51 | **<0.001** | 0.11 | 0.745 |
| **Eg** |  |  |  |  |  |  |
| rLN | 28.68 | **<0.001** | 10.55 | **0.001** | 1.09 | 0.298 |
| Ccore | 3.67 | 0.068 | 9.92 | **0.002** | 0.03 | 0.870 |
| Ccom | 48.79 | **<0.001** | 0.31 | 0.579 | 1.69 | 0.194 |
| **Lp** |  |  |  |  |  |  |
| rLN | 34.90 | **<0.001** | 9.85 | **0.002** | 1.65 | 0.201 |
| Ccore | 3.28 | 0.071 | 9.10 | **0.003** | 0.04 | 0.834 |
| Ccom | 50.48 | **<0.001** | 0.30 | 0.587 | 3.50 | 0.063 |

Eloc=local efficiency, Cp=Clustering Coefficient, FCS= functional connection strength, Eg=global efficiency, Lp=characteristic path length; rLN=reorganized language network, Ccore=core community, Ccom=compensatory community, Ccore2Ccom=connections from core community to compensatory community, Ccom2Ccore= connections from compensatory community to core community.

**Table S8.** Correlations between clinicopathological factors and significantly different topological indicators (PA vs. NC, p<0.05) across patients.

| Network Property | Topological  Indicator | Functional State | Grade | | Volume | | Duration | |
| --- | --- | --- | --- | --- | --- | --- | --- | --- |
|  |  |  | R | P | R | P | R | P |
| **Strength** |  |  |  |  |  |  |  |  |
| **rLN** |  |  |  |  |  |  |  |  |
|  | FCS | Rest | **-0.227** | **0.023** | **-0.207** | **0.039** | 0.022 | 0.825 |
|  |  | Task | -0.034 | 0.740 | 0.016 | 0.875 | 0.066 | 0.511 |
| **Ccore** |  |  |  |  |  |  |  |  |
|  | FCS | Rest | -0.119 | 0.238 | -0.164 | 0.104 | 0.021 | 0.836 |
|  |  | Task | -0.018 | 0.858 | -0.104 | 0.302 | 0.115 | 0.254 |
| **Integration** |  |  |  |  |  |  |  |  |
| **rLN** |  |  |  |  |  |  |  |  |
|  | Eg | Rest | **-0.217** | **0.030** | **-0.230** | **0.022** | 0.096 | 0.343 |
|  | Lp | Rest | 0.194 | 0.053 | 0.202 | 0.044 | -0.127 | 0.207 |
| **Ccore** |  |  |  |  |  |  |  |  |
|  | Eg | Rest | -0.106 | 0.296 | -0.188 | 0.062 | 0.043 | 0.674 |
|  |  | Task | 0.001 | 0.996 | -0.086 | 0.392 | 0.163 | 0.106 |
|  | Lp | Rest | 0.100 | 0.325 | 0.178 | 0.077 | -0.062 | 0.541 |
|  |  | Task | -0.026 | 0.800 | 0.038 | 0.704 | -0.137 | 0.174 |
| **Segregation** |  |  |  |  |  |  |  |  |
| **rLN** |  |  |  |  |  |  |  |  |
|  | Eloc | Rest | -0.186 | 0.063 | -0.187 | 0.063 | 0.127 | 0.207 |
| **Interaction** |  |  |  |  |  |  |  |  |
| **Ccom2Ccore** |  |  |  |  |  |  |  |  |
|  | FCS | Rest | -0.074 | 0.466 | -0.128 | 0.204 | 0.016 | 0.874 |
|  |  | Task | -0.026 | 0.798 | 0.042 | 0.679 | 0.048 | 0.639 |

PA=glioma patients, NC=normal controls, Eloc=local efficiency, Cp=Clustering Coefficient, FCS= functional connection strength, Eg=global efficiency, Lp=characteristic path length; rLN=reorganized language network, Ccore=core community, Ccom=compensatory community.

**Table S9.** Correlations between clinicopathological factors and behavioral performances across patients.

| Clinical Factor | MMSE | | Language | |
| --- | --- | --- | --- | --- |
|  | R | P | R | P |
| Grade | **-0.267** | **0.007** | **-0.379** | **<0.001** |
| Volume | -0.151 | 0.134 | **-0.200** | **0.046** |
| Duration | 0.076 | 0.453 | 0.072 | 0.474 |

**Table S10.** Correlations between significantly different LI indictors (PA vs. NC, p<0.05) and behavioral performances across patients.

| LI Indicator | | MMSE | | Language | |
| --- | --- | --- | --- | --- | --- |
| Network | Mask | R | P | R | P |
| **SN** |  |  |  |  |  |
|  | Frontal | **0.286** | **0.004** | **0.201** | **0.045** |
|  | Parietal | **0.259** | **0.009** | **0.206** | **0.039** |
|  | Temporal | 0.145 | 0.149 | 0.102 | 0.313 |
| **CEN** |  |  |  |  |  |
|  | Frontal | **0.271** | **0.006** | **0.212** | **0.034** |
|  | Parietal | 0.185 | 0.065 | **0.239** | **0.017** |

PA=glioma patients, NC=normal controls, CEN=central executive network, SN=salience network.

**Table S11.** Correlations between significantly increased activation (PA vs. NC, FDR-corrected, p<0.05) and behavioral performances across patients.

| Cluster Level | | MMSE | | Language | |
| --- | --- | --- | --- | --- | --- |
| Region | Side | R | P | R | P |
| Insula-anterior-4 | Left | 0.176 | 0.079 | 0.196 | 0.050 |
| Precuneus-1 | Left | -0.123 | 0.224 | -0.140 | 0.166 |
| Sup-Frontal-3 | Left | 0.057 | 0.572 | 0.081 | 0.426 |
| Fusiform-3 | Left | 0.068 | 0.504 | 0.042 | 0.681 |
| Vermis-4-5 | Left | -0.048 | 0.635 | -0.034 | 0.734 |
| Angular-3 | Left | -0.104 | 0.302 | -0.134 | 0.185 |
| Fusiform-4 | Right | -0.102 | 0.312 | -0.102 | 0.310 |
| Frontal-Sup-2 | Right | -0.051 | 0.614 | 0.045 | 0.658 |
| Frontal-Inf-Tri-1 | Right | -0.063 | 0.535 | 0.029 | 0.775 |
| Cerebelum-8 | Right | -0.058 | 0.564 | -0.071 | 0.484 |
| Insula-anterior-4 | Right | -0.080 | 0.429 | -0.034 | 0.740 |
| Postcentral-2 | Right | -0.155 | 0.123 | -0.171 | 0.089 |

PA=glioma patients, NC=normal controls.

**Table S12.** Correlations between the significantly topological indicators (PA vs. NC) and behavioral performances across patients.

| Network Property | Topological  Indicator | Functional State | MMSE | | Language | |
| --- | --- | --- | --- | --- | --- | --- |
|  |  |  | R | P | R | P |
| **Strength** |  |  |  |  |  |  |
| **rLN** |  |  |  |  |  |  |
|  | FCS | Rest | 0.069 | 0.496 | 0.124 | 0.220 |
|  |  | Task | -0.108 | 0.283 | 0.121 | 0.229 |
| **Ccore** |  |  |  |  |  |  |
|  | FCS | Rest | 0.002 | 0.981 | **0.200** | **0.046** |
|  |  | Task | -0.100 | 0.324 | 0.120 | 0.236 |
| **Integration** |  |  |  |  |  |  |
| **rLN** |  |  |  |  |  |  |
|  | Eg | Rest | 0.041 | 0.688 | 0.199 | 0.237 |
|  | Lp | Rest | -0.049 | 0.631 | -0.136 | 0.176 |
| **Ccore** |  |  |  |  |  |  |
|  | Eg | Rest | -0.014 | 0.893 | **0.219** | **0.028** |
|  |  | Task | -0.048 | 0.639 | 0.146 | 0.148 |
|  | Lp | Rest | -0.048 | 0.638 | **-0.270** | **0.007** |
|  |  | Task | 0.025 | 0.802 | -0.168 | 0.094 |
| **Segregation** |  |  |  |  |  |  |
| **rLN** |  |  |  |  |  |  |
|  | Eloc | Rest | 0.081 | 0.421 | 0.172 | 0.087 |
| **Ccore** |  |  |  |  |  |  |
|  | Cp | Task | -0.135 | 0.181 | -0.065 | 0.521 |
| **Interaction** |  |  |  |  |  |  |
| **Ccom2Ccore** |  |  |  |  |  |  |
|  | FCS | Rest | 0.103 | 0.306 | 0.129 | 0.200 |
|  |  | Task | 0.009 | 0.931 | 0.131 | 0.193 |

PA=glioma patients, NC=normal controls, Eloc=local efficiency, Cp=Clustering Coefficient, FCS= functional connection strength, Eg=global efficiency, Lp=characteristic path length; rLN=reorganized language network, Ccore=core community, Ccom=compensatory community.
